# The Arabidopsis PHD-finger protein EDM2 has multiple roles in balancing NLR immune receptor gene expression

**DOI:** 10.1371/journal.pgen.1008993

**Published:** 2020-09-14

**Authors:** Yan Lai, Xueqing Maggie Lu, Josquin Daron, Songqin Pan, Jianqiang Wang, Wei Wang, Tokuji Tsuchiya, Eric Holub, John M. McDowell, R. Keith Slotkin, Karine G. Le Roch, Thomas Eulgem

**Affiliations:** 1 Center for Plant Cell Biology, Institute of Integrative Genome Biology, Department of Botany and Plan Sciences, University of California at Riverside, Riverside, CA, United States of America; 2 College of Life Sciences, Fujian Agricultural and Forestry University, Fuzhou, Fujian, China; 3 Center for Infectious Disease and Vector Research, Institute of Integrative Genome Biology, Department of Molecular, Cell and Systems Biology, University of California at Riverside, Riverside, CA, United States of America; 4 Department of Molecular Genetics, The Ohio State University, Columbus, Ohio, United States of America; 5 Department of Plant Pathology, Physiology, and Weed Science, Virginia Tech, Blacksburg, VA, United States of America; 6 College of Bioresource Sciences, Nihon University, Kanagawa, Japan; 7 School of Life Sciences, University of Warwick, Wellesbourne campus, United Kingdom; 8 Donald Danforth Plant Science Center, St. Louis, Missouri, United States of America; 9 Division of Biological Sciences, University of Missouri, Columbia, Missouri, United States of America; University of California Davis, UNITED STATES

## Abstract

Plant NLR-type receptors serve as sensitive triggers of host immunity. Their expression has to be well-balanced, due to their interference with various cellular processes and dose-dependency of their defense-inducing activity. A genetic “arms race” with fast-evolving pathogenic microbes requires plants to constantly innovate their NLR repertoires. We previously showed that insertion of the *COPIA-R7* retrotransposon into *RPP7* co-opted the epigenetic transposon silencing signal H3K9me2 to a new function promoting expression of this *Arabidopsis thaliana* NLR gene. Recruitment of the histone binding protein EDM2 to *COPIA-R7-*associated H3K9me2 is required for optimal expression of *RPP7*. By profiling of genome-wide effects of EDM2, we now uncovered additional examples illustrating effects of transposons on NLR gene expression, strongly suggesting that these mobile elements can play critical roles in the rapid evolution of plant NLR genes by providing the “raw material” for gene expression mechanisms. We further found EDM2 to have a global role in NLR expression control. Besides serving as a positive regulator of *RPP7* and a small number of other NLR genes, EDM2 acts as a suppressor of a multitude of additional NLR genes. We speculate that the dual functionality of EDM2 in NLR expression control arose from the need to compensate for fitness penalties caused by high expression of some NLR genes by suppression of others. Moreover, we are providing new insights into functional relationships of EDM2 with its interaction partner, the RNA binding protein EDM3/AIPP1, and its target gene *IBM1*, encoding an H3K9-demethylase.

## Author summary

We previously found the *Arabidopsis thaliana* PHD-finger protein EDM2 to serve as a chromatin-associated factor controlling expression of the NLR-type immune receptor gene *RPP7*. EDM2 binds to the transposon-silencing signal H3K9me2 and affects levels of this epigenetic mark at various loci. By genome-wide profiling of transcript- and H3K9me2-levels we now found EDM2 to have a broader role in controlling NLR gene expression. In order to mitigate fitness costs caused by its promoting effects on *RPP7* expression and that of several other NLR genes, EDM2 seems to suppress expression of many additional members of this gene family. This observation is in line with multiple reports demonstrating the need for balanced expression of NLRs, which can substantially reduce overall plant fitness, but need to be present at certain minimal levels to confer sufficient immune protection. Our previous results demonstrated that the influence of EDM2 on *RPP7* expression was co-opted to this immune receptor gene by the insertion of an EDM2-controlled transposon. Here, we are providing additional examples for transposon-associated effects on NLR gene expression, suggesting that these mobile elements play an important role for NLR genes by equipping members of this rapidly evolving gene family with regulatory mechanisms needed for balanced expression.

## Introduction

Plant NLR (nucleotide-binding, leucine-rich repeat) genes encode sensitive immune receptors that can mediate specific recognition of microbial pathogens [1, 2]. Upon direct or indirect interactions with pathogen avirulence (*AVR*) gene products, these receptors induce a set of strong defense reactions, such as the hypersensitive response (HR), a form of local programmed cell death. A genetic “arms race” with fast-evolving pathogenic microbes requires plants to constantly innovate their NLR repertoires enabling them to maintain the capacity to recognize their microbial foes. Proper homeostasis of NLR activity is critical for their function [2, 3] and NLR expression is subject to strict regulation [4]. The ability of NLRs to trigger immunity is dependent on their dose [5, 6], while overexpression of NLR genes can result in autoimmunity and fitness penalties, such as reduced growth and impaired development of reproductive tissues [7, 8]. Various strategies have been proposed to mitigate the trade-off between NLR activity and fitness [9]. These include limiting expression of these immune receptors to certain times and tissues [9, 10] as well as tight genetic linkage to loci preventing their inadvertent activity [9, 11, 12]. Furthermore, in several plant species a micro-RNA-based network seems to prevent intolerably high levels of NLR expression [4, 13]. Nonetheless, due to continuous background activity of these immune receptors, a minimal fitness penalty seems unavoidable. Clearly, plants must be under strong selective pressures to evolve mechanisms enabling them to maximize their defense capacity, while limiting negative impact on their fitness. As a result, multiple regulatory steps affecting transcription as well as co/post-transcriptional processing and transcript turn-over strictly regulate levels of NLR transcripts [4]. However, evolutionary mechanisms enabling plants to rapidly evolve such mechanisms of tight NLR expression control are poorly understood.

We previously reported on the *Arabidopsis thaliana ENHANCED DOWNY MILDEW 2* (*EDM2)* gene, which is required for proper expression and function of *RPP7*, an NLR gene mediating strong resistance against the Hiks1 isolate of the biotrophic oomycete *Hyaloperonospora arabidopsidis* (*Hpa*; causal agent of Arabidopsis downy mildew) [14]. *EDM2* encodes a nuclear protein featuring 2 ½ repeats of an atypical PHD finger motif, several acidic domains, a plant G gamma-like-related (PGR) domain, an N6-adenine methyltransferase-like domain conserved in EDM2-like plant proteins (ELP domain) and a proline-rich C-terminal region [14–16]. EDM2 positively controls levels of RPP7-coding transcripts, which correlate with levels of immunity mediated by this NLR gene [14, 17, 18]. We also reported the identification of EDM2-interacting proteins, which include EMSY-like nucleosome remodeling factors and the WNK8 protein kinase [15, 17]. Besides this EDM2 has been shown to interact with the RNA binding proteins ASI1/IBM2 and EDM3/AIPP1 [19, 20]. We further found that EDM2 controls silencing of some transposable elements (TEs) by modulating levels of di-methylated lysine 9 of histone H3 (H3K9me2), a ubiquitous TE silencing signal in plants [21]. Besides being compromised in *RPP7*-mediated immunity, mutants of EDM2 exhibit several developmental phenotypes [15, 22], including retarded growth and abnormally shaped leaves, reminiscent of *cpr* or *cim* mutants [23, 24], which exhibit constitutively activated immunity. Trans-generational variability and instability of such phenotypes [21] suggested roles of EDM2 in epigenetic processes.

EDM2 affects levels of RPP7-coding transcripts by controlling alternative polyadenylation [18]. It promotes high levels of H3K9me2 at a TE-associated proximal polyadenylation site in the first *RPP7* intron, binds to H3K9me2-marked chromatin at this proximal polyadenylation site and suppresses its use, thereby promoting high levels of RPP7 transcripts that encode the full-length NLR protein. Loss of EDM2 function leads to pronounced accumulation of the alternative, non-coding ECL (exon 1-containing 5’ LTR terminated) RPP7 transcript, which is terminated/polyadenylated at the 5’ LTR of a retrotransposon inserted in the 1^st^ RPP7 intron. We further showed this EDM2- and H3K9me2-dependent alternative polyadenylation mechanism to be responsive to *Hpa* recognition and to dynamically adjust *RPP7* expression levels during the induction of immune responses.

Like H3K9me2, methylation of the DNA base cytosine at position 5 (5mC) is involved in the silencing of TEs. In plants, 5mC occurs at symmetrical GC and CHG motifs or asymmetrical CHH sites (H = any nucleobase, except G). While *de novo* cytosine methylation seems globally controlled in Arabidopsis by the RNA-directed DNA methylation pathway [25, 26], maintenance methylation of CHH and CHG sites is linked to H3K9me2 via CMT2 and CMT3, two cytosine methyltransferases which can bind to H3K9me2 [27, 28]. EDM2 also affects CHG methylation (5mCHG) [16, 21]. However, at *RPP7* and several TEs we found the effect of EDM2 on H3K9me2 to be substantially more pronounced compared to its effect on 5mCHG [18, 21]. We further found the directionality of EDM2-mediated changes of H3K9me2 and 5mCHG levels to depend on the local DNA sequence and/or chromatin context. For example, EDM2 has a suppressive effect on these marks at the *COPIA4* retrotransposon, while it promotes high levels of both silencing marks at the DNA transposon *Mu1* [21].

Genome-wide profiling in Arabidopsis by bisulfite-sequencing showed EDM2 to globally suppress 5mCHG in the bodies of hundreds of genes associated with heterochromatic repeat and TE sequences, thereby preventing these genes from being silenced by the spread of silencing marks [16]. Critical for this function appears to be the H3K9 demethylase IBM1, the expression of which is controlled by EDM2. Similar to its effect on *RPP7*, EDM2 binds to a heterochromatic region in a long *IBM1* intron where it suppresses premature transcript polyadenylation/termination, thereby promoting the synthesis of full length IBM1 mRNAs [16]. Besides *RPP7* and *IBM1*, mRNA-seq analysis identified 55 Arabidopsis genes possibly regulated by EDM2-mediated suppression of proximal polyadenylation at heterochromatic introns. In addition to *RPP7*, only one NLR gene, *RRS1*, is present in this set of putative EDM2 targets [16].

In the current study, we profiled in *edm2* mutant plants transcripts, and H3K9me2 by RNA-seq and ChIP-seq, respectively, to expand our understanding of genome-wide roles of EDM2 in Arabidopsis. We observed effects of EDM2 on H3K9me2 and/or transcript levels of at least 59 NLR genes. In most cases, EDM2 is suppressing their expression, while it promotes expression of only a small number of NLR genes. Thus, EDM2 appears to be a global regulator of NLR genes, balancing the expression of members of this gene family by prioritizing some family members at the expense of others.

Our results further indicate a major role of EDM2 in the regulation of TEs, as it affects H3K9me2 and/or transcript levels of at over 2,000 TE loci. By ChIP-seq we identified multiple cases where binding of an epitope-tagged EDM2 version is centered on a TE immediately adjacent to or residing within an NLR gene. We found EDM2 to influence expression of the NLR gene *RPP4* in a manner similar to *RPP7*, as this PHD finger protein binds to a TE closely associated with *RPP4* and promotes the synthesis of full-length RPP4 transcripts. Besides *RPP7* and *RPP4* we provide additional examples for TE-linked effects on NLR expression suggesting that TE insertions have repeatedly recruited regulatory mechanisms into the context of Arabidopsis NLR genes. Together with other recent reports [29–31], our data strongly support that TEs can play a major role in NLR evolution by providing the “raw material” for new gene regulatory mechanisms. Consistent with this view, we observed a statistically significant association of TEs with NLR loci in the Arabidopsis genome. This important function, however, is not limited to EDM2-controlled NLRs, as both EDM2-dependent and EDM2-independent NLRs are significantly associated with transposons.

## Results

### Genome-wide profiling of EDM2-mediated effects on H3K9me2 and transcripts

We profiled H3K9me2 by chromatin immunoprecipitation (ChIP)-seq in the *edm2-2* mutant and its parental wild type accession Col-0 (WT) using an anti-histone H3K9me2 antibody. To evaluate the quality of our data, we also performed ChIP-seq with input DNA (sonicated chromatin) and anti-histone H3 C-terminal (H3C) antibody-immunoprecipitated DNA from *edm2-2* and WT, respectively. For all types of ChIP-seq libraries two independent replicates were sequenced.

As shown in S1 Fig, levels of H3C and input chromatin are consistently low and no peaks were detected for these tracks. High levels of Spearman correlation were observed for each pair of ChIP-seq replicates (R = 0.982 for WT libraries and R = 0.979 for *edm2-2* libraries, S2 Fig). Taken together, these results indicate that our ChIP-seq data are highly reproducible with minimal background noise.

**Fig 1:**
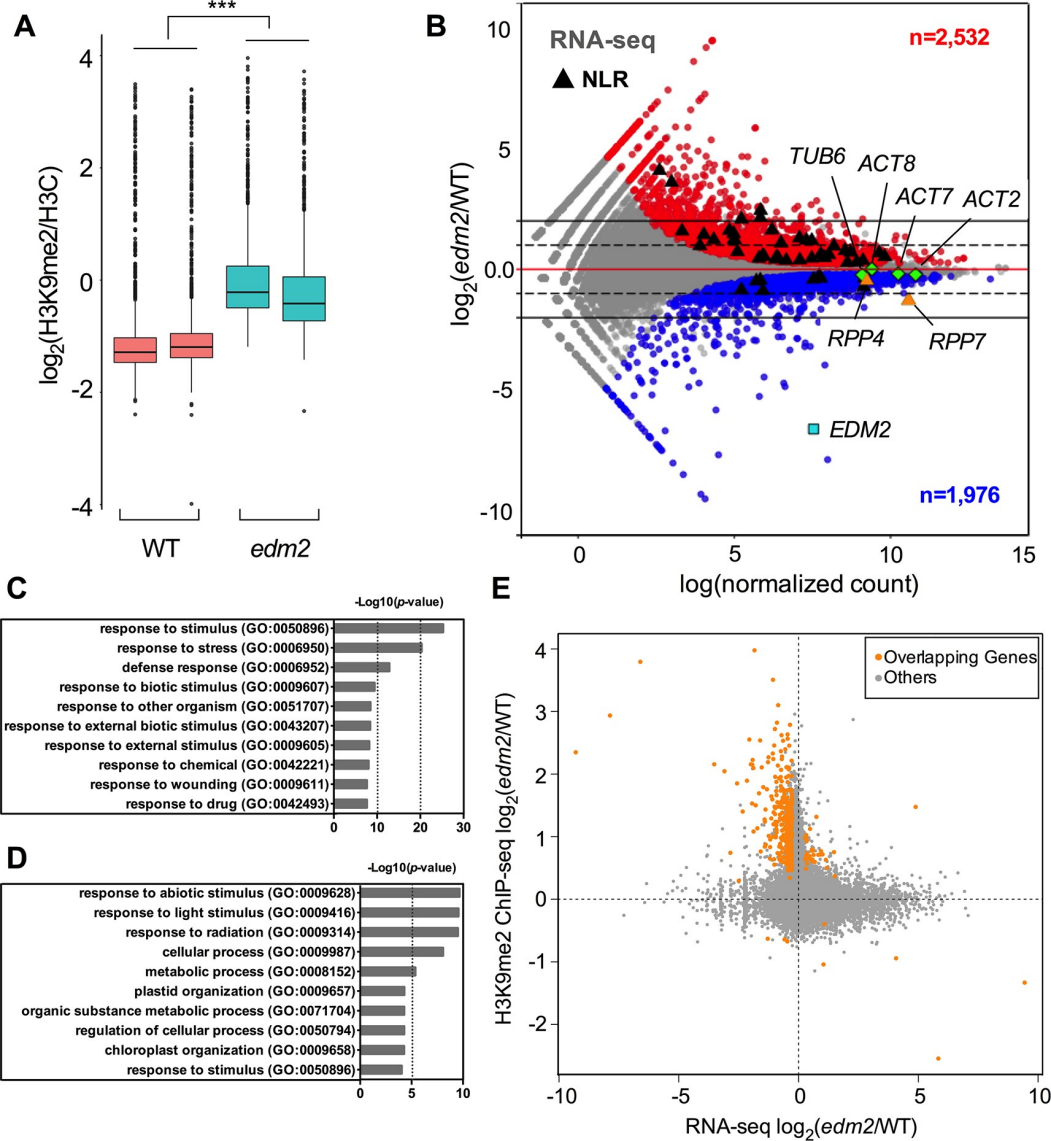
EDM2 affects genome-wide H3K9me2 and transcripts. **(A)** Box plot showing normalized H3K9me2 levels relative to histone H3 distribution for significantly affected genes (*P* adjust value < 0.05) in each replicated of WT ChIP-seq sample (red boxes) and *edm2-2* ChIP-seq sample (turquoise boxes). Statistical significance of differences between WT and *edm2-2* is determined using combined gene counts of replicates and by Wilcoxon signed rank test. ***: *P*-value < 2.2e-16. **(B)** The minus-average (MA) plot representing differential expressed genes in *edm2*/WT. Displayed are fold change values (y-axis) against expression levels (x-axis). Genes exhibiting significant differential expression changes between WT and *edm2-2* (*P* adjust value < 0.05) are colored in red and blue, where red marked *edm2-2* up-regulated genes and blue marked the *edm2-2* down-regulated genes. NLR genes with *P* adjust value < 0.05 are represented by black triangles. *ACTIN2* (*ACT2*), *ACTIN7* (*ACT7*), *ACTIN8* (*ACT8*) and *TUBULIN6* (*TUB6*) are used as benchmarks for differential expression analysis and are labeled in green. **(C and D)** Significantly enriched GO (Gene Ontology) terms with *P* < 0.05 for the top 10 categories of *edm2-2* up-regulated genes **(C)** and down-regulated genes **(D)**. **(E)** Scatterplot showing the correlation of transcript levels versus H3K9me2 levels of genes differentially expressed and H3K9-dimethylated in *edm2-2* compared to WT.

For each annotated transcriptional unit (Araport11), relative H3K9me2 levels were calculated and compared between *edm2-2* and WT. For 2,082 genes and 1,736 TEs we observed significant H3K9 methylation differences between *edm2* and WT (S1 and S2 Tables). A majority of the genes that showed significant changes exhibit H3K9 hyper-dimethylation in *edm2-2* relative to WT (97.84%) indicating a major role of EDM2 in genome-wide suppression of H3K9me2 (Fig 1A), whereas a smaller number of significant changes represent H3K9 hypo-dimethylation in *edm2* relative to WT (Fig 1A and S1 Table). This is consistent with previous results on the effects of EDM2 on CHG methylation [16], a mark which is often directly correlated with H3K9me2 [32].

To investigate the genome-wide impact of EDM2 at the transcript level, we compared RNA-seq-generated transcript profiles between *edm2-2* and WT plants. Reads were mapped to each annotated transcriptional unit (Araport11). High levels of Spearman correlation were observed between biological replicates (S2 Fig). Relative transcript levels (normalized per million mapped reads) reflecting the abundance of all reads mapping to the respective transcriptional units were calculated and compared between *edm2-2* and WT. We refer to genes showing significant differences in transcript levels in these comparisons as differentially expressed and either up-regulated or down-regulated. Further below we are discussing alternative polyadenylation as a mechanism affecting expression of a small subset of EDM2-controlled genes. For these genes differences in transcript levels between *edm2-2* and WT plants are mainly limited to certain sub-regions (e.g. 3’ ends) not necessarily resulting in significant differences when entire transcriptional units are considered. Thus, some of them are not included in the sets of up- or down-regulated genes we are discussing here.

A total of 4,508 protein-encoding genes exhibited significant differential expression between *edm2-2* and WT including 2,532 genes (56%) that were up-regulated and 1,976 genes (44%) that were down-regulated in *edm2-2* relative to WT (Fig 1B and S3 Table). Gene ontology analysis showed that in terms of biological processes, genes with higher transcript levels in *edm2-2* (genes that are suppressed by EDM2) are highly significantly associated with diverse stress responses, including defense responses and biotic stimuli (Fig 1C), while genes exhibiting reduced transcript levels in this mutant (genes that are positively regulated by EDM2) are strongly associated with abiotic stress responses and cellular or metabolic processes (Fig 1D).

Of 369 genes that exhibit significant changes in both H3K9me2 and transcript levels in *edm2-2* compared to WT, 323 are down-regulated in *edm2-2* and show at the same time higher H3K9me2 levels in this mutant (Fig 1E). This suggests a causal connection between both effects supporting that EDM2 promotes transcription of these genes by suppressing their H3K9me2. Gene ontology analysis showed that in terms of biological processes, these 323 genes are strongly enriched for diverse metabolic functions (S3 Fig).

**Fig 2:**
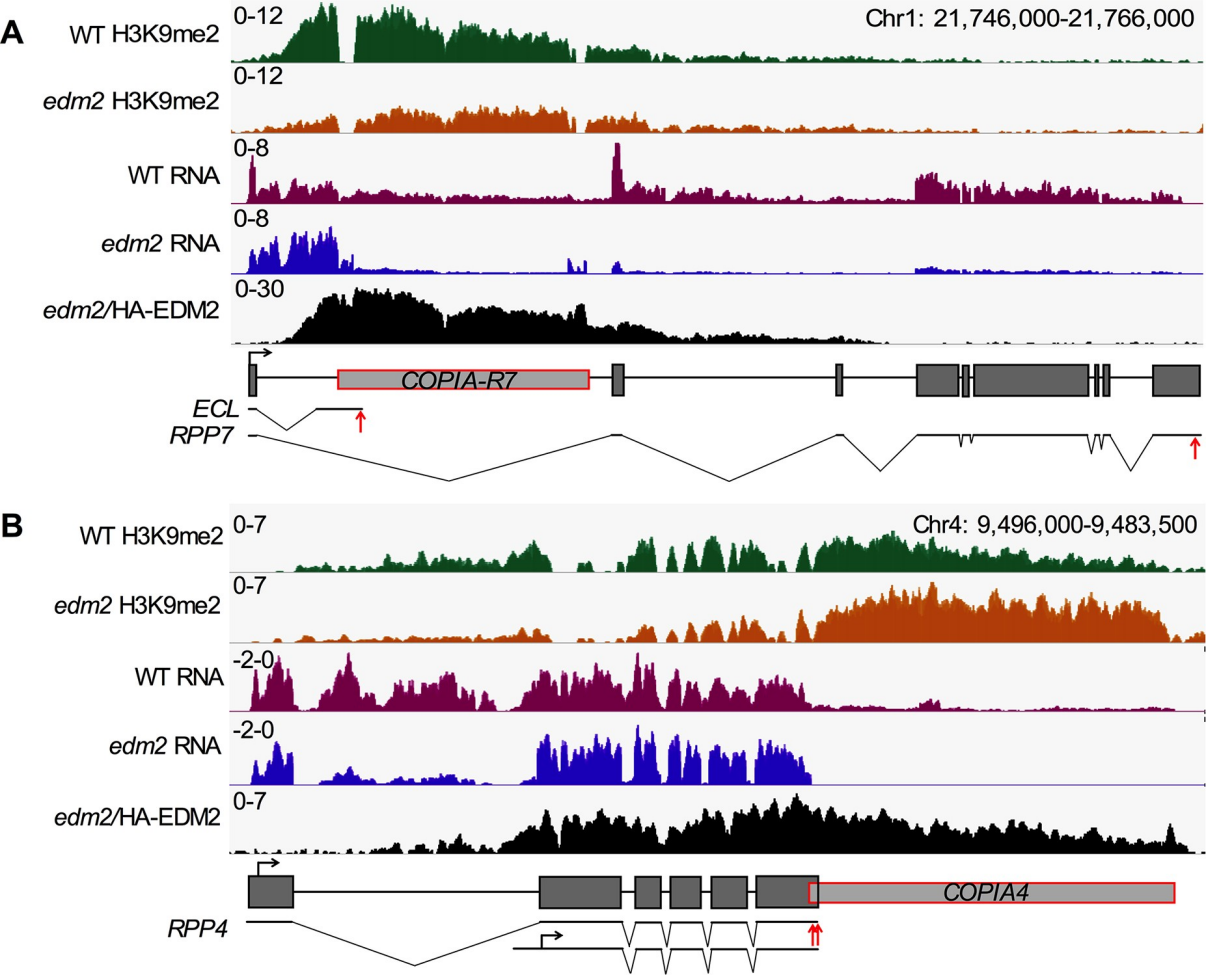
EDM2 is associated with *RPP7* and *RPP4*, and affects H3K9me2 and transcript levels at these loci. **(A and B)** Genome browser view of H3K9me2 ChIP-seq, RNA-seq and HA-tagged EDM2 ChIP-seq at *RPP7* **(A)** and *RPP4* **(B)**. Y*-*axis represents coverage values (normalized per million mapped reads). Schematic representations of the *RPP7* and *RPP4* loci with individual RNA transcript isoforms are shown at the bottom. Transposons are represented by grey boxes framed in red and genic exons by black-framed grey boxes. ECL: “Exon 1-containing 5’LTR terminated” non-coding transcript resulting from proximal polyadenylation/transcript termination at *RPP7*. Red vertical arrows indicate the polyadenylation sites and horizontal black arrows indicate transcription start sites.

Our H3K9me2 ChIP-seq and RNA-seq results are consistent with our previous findings at the characterized EDM2 target locus *RPP7*/*COPIA-R7* (Fig 2A and S4 Fig) [18]. Levels of H3K9me2 are high in WT at *COPIA-R7* and the *RPP7* intron 1 segment upstream of this TE, which templates for the non-coding ECL transcript. H3K9me2 levels in this area, however, are reduced in *edm2-2* plants. Furthermore, in *edm2-2* transcripts representing the ECL-templating area of *RPP7* (comprising the first *RPP7* exon and parts of its first intron) are highly expressed, while transcripts downstream of *COPIA-R7*, including the entire *RPP7* coding sequence, are strongly reduced. These results confirm our previously postulated role of EDM2 in promoting the synthesis of full-length RPP7 transcripts and suppressing proximal polyadenylation/transcript termination at the 5’LTR of *COPIA-R7*.

### EDM2 has a broad role in suppressing NLR transcript levels

Our H3K9me2 ChIP-seq and RNA-seq results revealed several interesting trends. Firstly, a large set of NLR genes (including *RPP7*) is affected by EDM2. In *edm2-2* compared to WT a total of 59 of 165 annotated Arabidopsis NLR loci show a significant change of their H3K9me2 and/or transcript levels (S4A and S4B Table). This includes the known functional *R* gene *RPP4*, which like *RPP7* exhibits H3K9 hypo-dimethylation and a mild (but significant) reduction of transcript levels in *edm2-2* (Fig 2B and S5 Fig). *RPP4* is a member of the Col-0 *RPP5* NLR cluster [33]. Another member of this cluster, AT4G16900, also belongs to the set of 59 EDM2-affected NLR genes.

**Fig 3:**
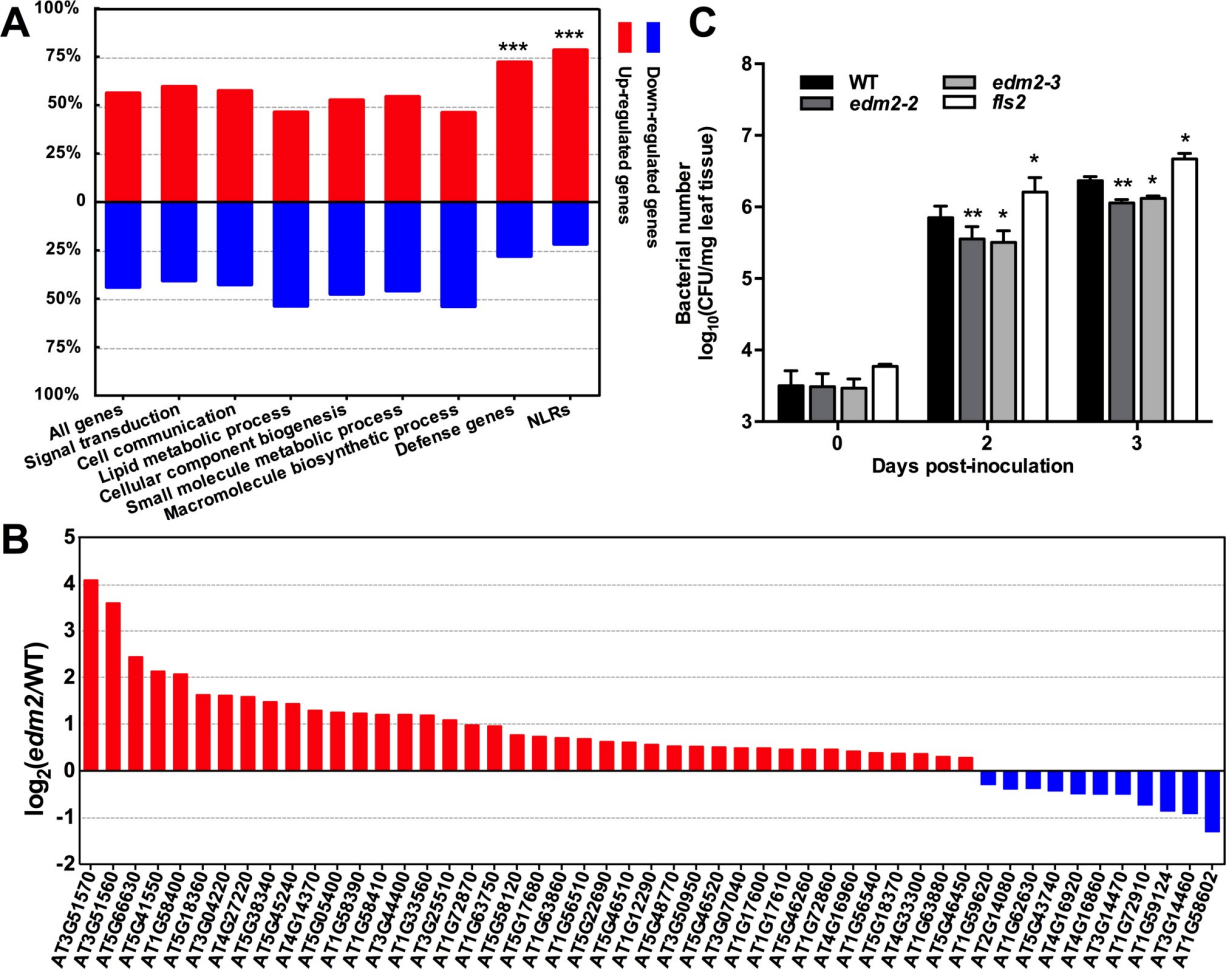
EDM2 suppresses transcript levels of many NLRs and basal defense. **A.** Distribution of all genes, defense genes, NLR genes and genes of several other functional categories (defined by enriched GO terms) that show significant transcript level changes in *edm2-2* compared to WT. Besides defense genes and NLRs, only non defense-associated categories with at least 130 genes differentially expressed in *edm2-2* compared to WT were considered. *χ*^2^ test of independence was performed to detect significant differences between actual and expected equal distribution (50% up-regulated and 50% down-regulated genes). ***: *P*-value <0.001. **B.** Transcript level changes of EDM2-affected NLR genes. Only NLR genes with significant transcript level differences between *edm2-2* and WT are included. **C.** Defense phenotype of *edm2* mutants infected with *Pseudomonas syringae* pv. *tomato* DC3000 (*Pst* DC3000). Bacterial multiplication was monitored at 3 hrs(0), 2 days post inoculation (dpi) and 3 dpi with a 2 ×10^8^ cfu/mL bacterial suspension. Error bars represent SEM for three biological replicates. *fls2* (SALK_141277) seedlings were used as a susceptible control. Statistical significance was determined by Student’s *t*-tests. *: *P*-value <0.05. **: *P*-value <0.01.

As shown in Fig 3A, the sets of all genes that are significantly up- or down-regulated in *edm2-2* compared to WT are nearly equally large with 56% up-regulated genes and 44% down-regulated genes in this mutant. This general trend also applies to most functional categories of differentially expressed genes (Fig 3A). However, the role of EDM2 is significantly shifted towards a broad function in suppression of NLR gene expression, as the majority of differentially expressed NLR genes (78%) are up-regulated, while only 22% are down-regulated in *edm2-2* compared to WT (Fig 3A and 3B). Overexpression of NLRs is known to result in constitutive activation of defense responses [4, 7, 8]. Consistent with this, we previously found *edm2* mutant plants to exhibit moderately elevated levels of basal resistance against the virulent isolate Noco2 of the pathogenic oomycete *Hyaloperonospora arabidopsidis* [15, 17]. We also observed *edm2* mutants to show enhanced resistance against the virulent DC3000 strain of the bacterial pathogen *Pseudomonas syringae* (Fig 3C). As a likely consequence of the collective over-expression of numerous NLR genes in *edm2-2*, the majority of EDM2-affected genes annotated as defense-associated are also up-regulated in *edm2-2* compared to WT (Fig 3A). These include genes of key plant immune regulators, such as many WRKY and ERF transcription factors (S4C and S4D Table). As overexpression of NLR genes is also known to reduce fitness and to impair development of plants [4, 7, 8], the stunted nature and morphological abnormalities of *edm2* mutants [21] are also likely, at least partially, a consequence of the collective up-regulation of NLR transcripts in this mutant.

Collectively our observations show that EDM2 plays a role in balancing transcript levels of NLRs. In the case of *RPP7* and *RPP4*, as well as several others, it seems to promote their expression to levels required for full pathogen resistance (see below), while in most other cases, EDM2 may limit their expression to levels that are non-detrimental for proper plant development and fitness, thereby serving as a suppressor of constitutive defense activation.

### EDM2 affects over 2,000 TEs

**Fig 4:**
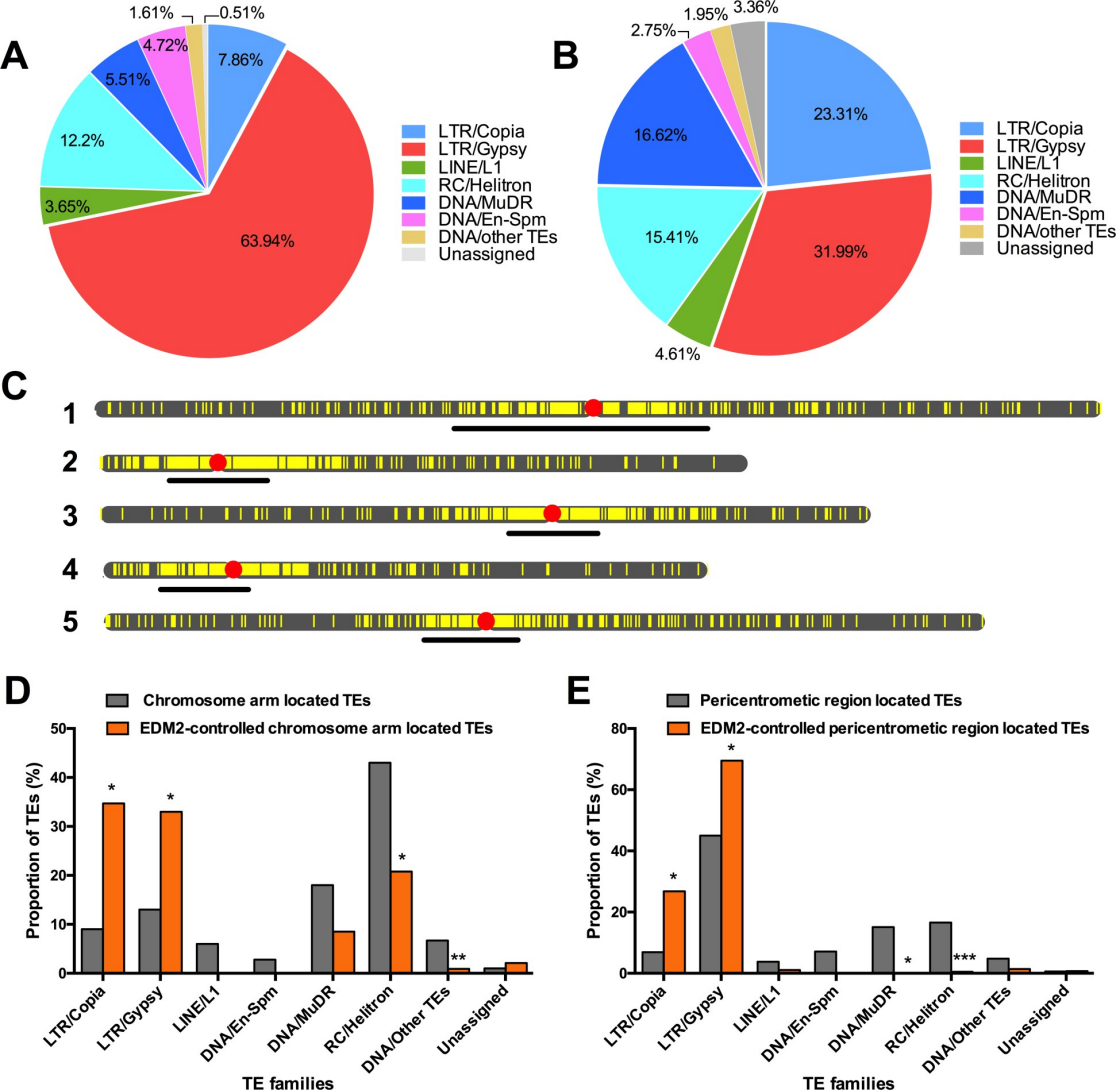
EDM2 affects H3K9me2 and transcript levels at hundreds of TEs. **(A and B)** Pie charts representing types of TEs differentially affected by EDM2 (*edm2-2* vs. WT) in H3K9me2 ChIP-seq **(A)** or RNA-seq data **(B)** sets. **(C)** Chromosomal locations of TEs significantly affected by EDM2 in H3K9me2 ChIP-seq and/or RNA-seq data. The chromosome map tool (https://www.arabidopsis.org/jsp/ChromosomeMap/tool.jsp) was used to define the locations of TEs. Vertical yellow lines represent individual TEs. Horizontal bars mark centromeric and pericentromeric heterochromatic areas. **(D and E)** Proportion of EDM2-controlled TEs compared to all TEs located either in the chromosomal arms **(D)** or pericentromeric regions **(E)**. Statistical significance was determined by Fisher exact test. *: *P*-value <0.05. **: *P*-value <0.01. ***: *P*-value <0.001.

Our ChIP-seq data further indicate that EDM2 affects H3K9me2 levels of 1,736 TEs (out of 31,219 analyzed TEs; S2 Table). Of these, 1,124 TEs exhibit higher H3K9me2 levels in *edm2-2* compared to WT, while 612 TEs show the opposite behavior. The vast majority of EDM2-affected TEs are retrotransposons, such as Gypsy (63.94%), Copia (7.86%) or non-LTR/LINE (3.65%) elements (Fig 4A). Although a much smaller number of TEs showed significant changes in transcript levels (241 up-regulated and 243 down-regulated in *edm2-2*; Fig 4B and S5 Table), we still observed that the majority of EDM2-controlled TEs are retrotransposons (Gypsy (31.99%), Copia (23.31%) and non-LTR/LINE (4.61%) elements). Taken together, we observed regulatory effects of EDM2 on H3K9me2 and/or transcript levels of a total of 2,144 TEs.

As shown in Fig 4C, the majority of all 2,144 EDM2-controlled TEs are located in the pericentromeric regions. Furthermore, EDM2 has a clear preference for Copia- and Gypsy retrotransposons. In comparison to all TEs located either in the chromosomal arms or pericentromeric regions, Copia and Gypsy TEs are significantly enriched in the set of TEs affected by EDM2 in their transcript and H3K9me2 levels (Fig 4D and 4E). Taken together, our results highlight a major role of EDM2 in controlling retrotransposons, especially for Copia and Gypsy TEs localized in chromosomal arms and pericentromeric regions.

### Several NLR genes and TEs are direct *in vivo* targets of EDM2

Previous studies identified *RPP7* and *IBM1* as direct EDM2 targets [16, 18]. To identify additional direct EDM2 targets, we performed ChIP-seq with a transgenic *edm2-2* complementation line expressing cDNA-encoded EDM2 fused to the HA epitope-tag at its N-terminus and driven by the native *EDM2* promoter (*E2pro*:*HA-E2c*). *RPP7*-mediated resistance against *Hpa*Hiks1 and wild-type *RPP7* mRNA levels are restored in this line [18]. RNA-seq with this line also confirmed that near wild type transcript levels are restored at *RPP7* and *RPP4* as well as other loci of interest for this study (S4 and S5 Figs). Specificity of our ChIP procedure for HA-EDM2 was validated by mass-spectroscopy (S6 Fig). By mapping unique reads to transcribed units we observed significant levels of association of HA-EDM2 with 32 genes (S6A Table). Besides *RPP7*, genes directly targeted by HA-EDM2 include the two Col-0 *RPP5* NLR cluster members *RPP4* and AT4G16900 and several loci within the *RPP7 NLR* cluster (Fig 2 and S6 Table) as well as the *EDM2* locus itself (S7A Fig) [16]. Consistent with previous observations, we also observed weak binding of HA-EDM2 to a heterochromatic region of *IBM1* intron 7 (S7B Fig) [16].

Using non-uniquely mapped reads, we observed direct binding of HA-EDM2 to at least 46 TEs (S6B Table), including *COPIA-R7* (AT1TE71775) and *COPIA4* (AT4TE42860). Among those, we also observed direct association of HA-EDM2 with the retrotransposons AT1TE12295 and AT3TE06550 (S8 Fig). Similar to the situation at *COPIA-R7*/*RPP7*, these two TEs are embedded in long introns of genes (AT1G11270 and AT3G05410, respectively) and feature high levels of EDM2-dependent H3K9me2 (S2 Table). In *edm2-2* the associated genes exhibit reduced levels of transcripts in exons downstream from the respective TEs, indicative of EDM2-mediated alternative polyadenylation control (S3 Table and S8 Fig), as also previously reported [16].

### EDM2 promotes synthesis of full-length transcripts of the Col-0 *RPP5* NLR cluster member *RPP4*

Our genome profiling data indicated effects of EDM2 on H3K9me2 and transcript levels at *RPP4*, an NLR gene present in the Col-0 *RPP5* cluster. In addition, in *E2pro*:*HA-E2c* plants HA-EDM2 is associated with chromatin of this NLR gene and the directly adjacent *COPIA4* retrotransposon (Fig 2B).

*RPP4* is a functional *R* gene that mediates race-specific disease resistance of the Arabidopsis Col-0 accession against the *Hpa* isolate Emoy2 [34]. The Col-0 *RPP4* allele consists of six exons. Downstream from this NLR gene is the *COPIA4* retrotransposon. By 3’RACE we found the 3’UTR of this exon to overlap with the 5’LTR of *COPIA4*, which harbors the most distal and several proximal alternative polyadenylation sites for RPP4 transcripts (S9 Fig). The termination of RPP4 transcripts at the 5’LTR of *COPIA4* is reminiscent of the termination of ECL transcripts at *RPP7/COPIA-R7*, as in both cases the 5’LTR of a retrotransposon inserted into the context of an NLR gene serves as a functional polyadenylation site.

**Fig 5:**
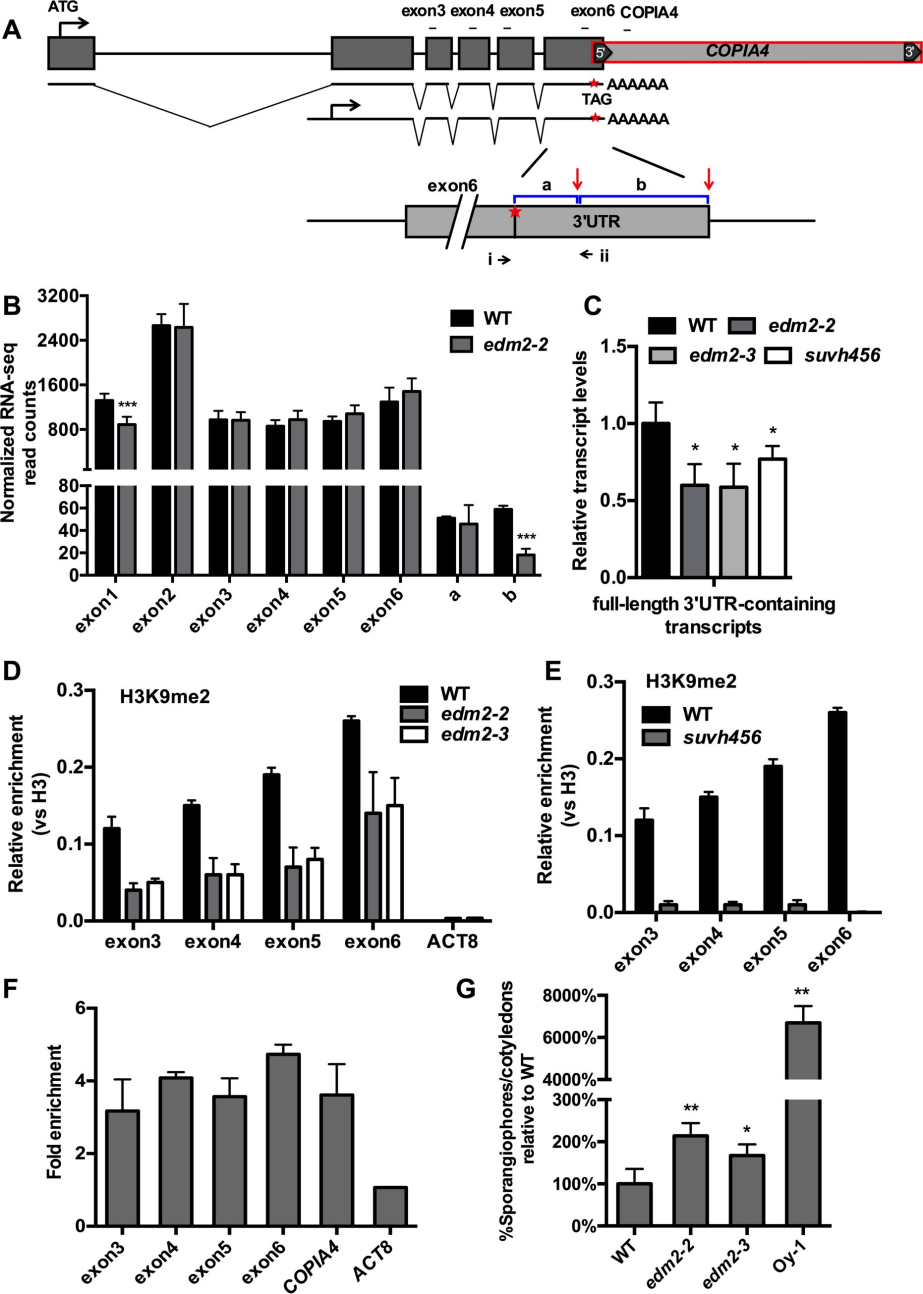
EDM2 controls alternative transcript polyadenylation at the *RPP4* locus and affects function of this NLR gene. **(A)** Schematic representation of *RPP4* with alternative RNA transcript isoforms. The termination codon (TAG) is marked by red star. Red vertical arrows represent polyadenylation sites determined by 3’RACE. Areas a (polyadenylation site #5 - #6) and b (polyadenylation site #6 - #8) are marked by blue horizontal lines and represent regions used for read counts shown in (B). Black horizontal arrows represent PCR primers used for qRT-PCR (i and ii) in (C). Regions amplified by qPCR for each exon in (D), (E) and (F) are represented by black horizontal bars. **(B)** RNA-seq read counts for *RPP4* exons1–6 and 3’UTR areas in WT and *edm2-2*. Exon1 contains 5’UTR and exon6 contains 3’UTR sequences. The coordinates for area a and b are 9488547–9488584 and 9488463–9488546, respectively. The y-axis represents normalized read counts calculated by DESeq2 in R. Error bars represent SD from three biological replicates. ***: *P*-adjusted value <0.001. **(C)** Levels of full-length 3’UTR-containing transcripts measured by qRT-PCR. Error bars represent SD from three biological replicates. Statistical significance was determined by Student’s *t*-tests. *: *P*-value <0.05. **(D)** Levels of H3K9me2 determined by ChIP-qPCR in WT, *edm2-2* and *edm2-3*. H3K9me2 levels were normalized to the total histone H3 levels. *ACTIN8* (ACT8) served as a control locus. Error bars represent SEM for two biological replicates each with three technical replicates. **(E)** Levels of H3K9me2 measured by ChIP-qPCR in WT and *suvh456*. H3K9me2 levels were normalized to the total histone H3 levels. Error bars represent SEM from two biological replicates each based on three technical replicates. **(F)** Levels of HA-EDM2 at *RPP4* in *E2pro*:*HA-E2c* line. *ACTIN8* (ACT8) served as a control locus. Fold enrichment values were measured by ChIP-qPCR relative to each area in WT. Error bars represent SEM from three biological replicates each based on three technical replicates. **(G)** Susceptibility phenotype of *edm2* mutants to the *Hyaloperonospora arabidopsidis* isolate Emoy2. Numbers of sporangiophores per cotyledons of WT, *edm2-2*, *edm2-3* and Oy1 lines 7 days post infection (dpi) with spores of the *RPP4*-recognized *Hpa-*Emoy2. Error bars represent SEM for five biological replicates, each replicate including at least 100 cotyledons. Statistical significance was determined by Student’s *t*-tests. *: *P*-value <0.05. **: *P*-value <0.01.

The ChIP-seq and RNA-seq results are consistent with our previous observation that in *edm2* mutants, H3K9me2 levels are substantially increased and transcripts undetectable at *COPIA4* [21]. Detailed inspection of our RNA-seq data suggested the existence of at least one proximal polyadenylation site in the *RPP4* 3’UTR region (Fig 2B, Fig 5A and 5B and S9D Fig). In *edm2-2* the presence of transcripts mapping to the *RPP4* 3’UTR abruptly stops in this area, while their levels are substantially reduced, but still clearly detectable, in WT. Transcripts mapping further downstream are continuously detectable throughout the 3’ portion of this exon and the entire *COPIA4* region in WT, but absent in *edm2-2*. By qRT-PCR we confirmed that levels of transcripts representing the complete sequence of *RPP4* full-length 3’UTR containing transcript, are significantly reduced in two independent *edm2* mutant alleles (*edm2-2* and *edm2-3*) compared to WT (Fig 5A and 5C). We further identified by 3’RACE multiple clustered proximal polyadenylation sites inside exon 6 and its 3’UTR region in both WT and *edm2-2* transcripts (S9B and S9D Fig). S9D Fig shows that the drop of read counts aligns with the location of the sixth of eight polyadenylation sites we determined by 3’RACE. Use of this proximal polyadenylation site is clearly suppressed by EDM2 resulting in a reduction of reads uniquely mapping to the area immediately downstream from it in *edm2-2* (area b stretching from polyadenylation site #6 to #8 in Fig 5B). The area immediately upstream from polyadenylation site #6 (area a, stretching from alternative polyadenylation site #5 to #6) does not exhibit a difference in read counts between WT and *edm2-2* (Fig 5A and 5B).

At *RPP7*, enhanced levels of premature polyadenylation in *edm2* mutants are causally linked to a reduction of H3K9me2 at its proximal polyadenylation site in the *COPIA-R7* 5’LTR [18]. Consistent with this, our H3K9me2 ChIP-seq results suggested in *edm2-2* a reduction of H3K9me2 levels over the entire *RPP4* locus relative to WT (Fig 2B). Using two independent *edm2* alleles we confirmed this for exons 3–6 by H3K9me2-ChIP-qPCR (Fig 5D). We also observed for *RPP4* a causal relationship between H3K9me2 levels and the extent of proximal polyadenylation. In the H3K9-methylase deficient *suvh456* triple mutant [35], where H3K9me2 is nearly undetectable at *RPP4* exons 3–6 (Fig 5E), levels of full-length 3’UTR containing transcripts are clearly reduced compared to WT and similar to *edm2* mutants (Fig 5C).

We further confirmed binding of HA-EDM2 to the *RPP4* region using ChIP-qPCR in the *E2pro*:*HA-E2c* line. As implied by our ChIP-seq data (Fig 2B), we detected clear enrichment of HA-EDM2-associated chromatin in several areas of *RPP4*, including exon 3–6 and *COPIA4* (Fig 5F).

Taken together our results show that EDM2 and TE-associated H3K9me2 have partially similar roles in expression control of *RPP4* and *RPP7*. EDM2 binds to H3K9me2-marked chromatin at *COPIA4*. H3K9me2 and binding of EDM2 seem to have spread from *COPIA4* into adjacent genic regions of *RPP4*, where they suppress proximal transcript polyadenylation, while promoting the production of full-length transcripts. Thus, the previously demonstrated recruitment of EDM2 into the regulatory context of *RPP7* polyadenylation control by insertion of a TE [18] is unlikely to be a unique case, highlighting the potential for related molecular processes to provide new regulatory mechanisms to NLR genes. Our results defined distinct proximal alternative polyadenylation sites in the overlap between *RPP4* and *COPIA4*, demonstrated EDM2/H3K9me2-dependent differential use of at least one of them and provided evidence for direct binding of EDM2 to this area.

### EDM2-deficient Arabidopsis lines exhibit reduced *RPP4*-mediated immunity

Our RNA-seq data suggested the impact of EDM2 on *RPP4* expression to be complex. Besides suppressing proximal polyadenylation in the 3’UTR, EDM2 seems to have additional effects on RPP4 transcripts. Our RNA-seq data revealed a significant reduction of RPP4 exon 1 transcript levels in *edm2-2* compared to WT (Figs 2B, 5A and 5B). No significant transcript level difference was observed for exons 2–6 (Fig 5B). This observation is consistent with the annotation of an alternatively initiated RPP4 transcript (TAIR10, Araport 11) bearing its transcription start site (TSS) in intron 1 slightly upstream from exon 2 (Figs 2B and 5A). Although we did not confirm this TSS, the existence of alternative TSSs directing expression of transcripts starting with exon 2 sequences is further supported by TSS-sequencing data from Kindgren et al. (S9E Fig) [36]. The N-terminally truncated RPP4 protein predicted to be encoded by such distally initiated transcripts lacks its entire TIR domain (S9A and S9C Fig). Thus, EDM2 may promote increased expression of exon 1- containing full length-transcripts encoding RPP4 proteins with the N-terminal TIR domain. Absence of this domain may compromise the immune receptor function of RPP4. Consistent with this, we found *edm2* mutants to exhibit a moderate (but significant) reduction of disease resistance in Arabidopsis cotyledons against the *RPP4* cognate *Hpa* isolate Emoy2 (Fig 5G). Likely, this effect is directly causally linked to mis-regulation of *RPP4* in *edm2* mutants and not due to some unspecific (or pleiotropic) effects. This view is supported by the fact that the NLR genes *RPP2A* and *RPP2B*, which are not affected in their expression in *edm2-2*, remain fully functional in this mutant. The NLR pair RPP2A and RPP2B is know to mediate in Col accessions of Arabidopsis immunity against the *Hpa* isolate Cala2 [37]. In our RNA-seq experiments we did not observe for *RPP2A* and *RPP2B* significant differences of reads mapped to their transcriptional units between *edm2-2* and Col-0. Neither does close inspection of genome browser views of RNA-seq data for both genes hint at minor local differences in RNA expression/processing (S9F and S9G Fig). Consequently, resistance against *Hpa*Cala2 has been reported not to be affected in *edm2* mutant plants [38].

### EDM2 affects additional members of the Col-0 *RPP5*, *RPP7* and *RPP1* NLR clusters

**Fig 6:**
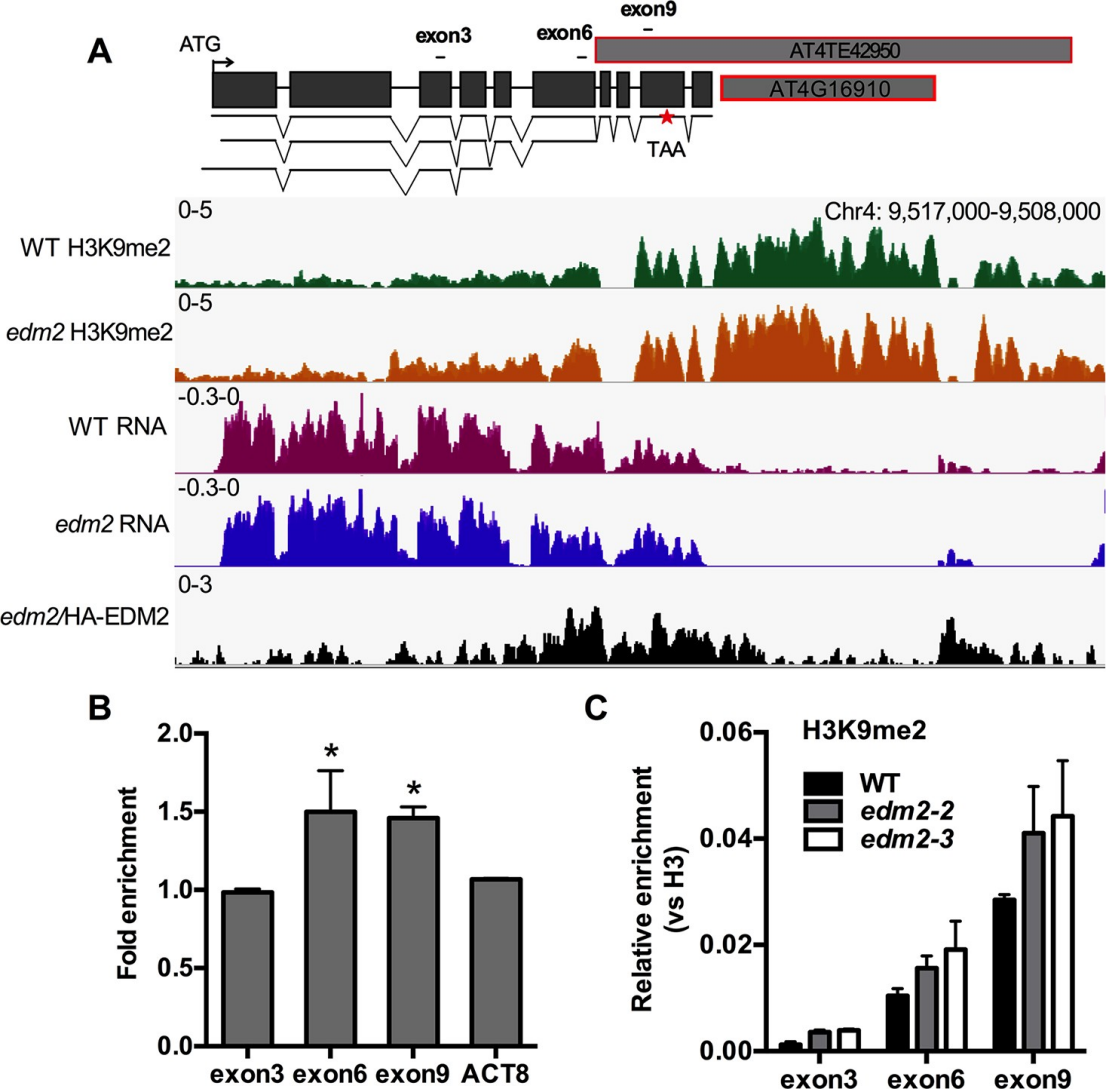
EDM2 affects AT4G16900. **(A)** Genome browser view of normalized H3K9me2 ChIP-seq, RNA-seq and HA-tagged EDM2 ChIP-seq at the AT4G16900 locus. The y*-*axis represents coverage values (normalized per million mapped reads). Schematic representations of the AT4G16900 locus with individual RNA transcript isoforms are shown in the top. The stop codon (TAA) is marked by a red star. Regions amplified by qPCR for each exons are represented by horizontal bars. **(B)** Levels of HA-EDM2 at AT4G16900 in the *E2pro*:*HA-E2c* line. *ACTIN8* (ACT8) served as a control locus. Fold enrichment values were measured by ChIP-qPCR relative to each respective area in WT. Error bars represent SEM for two biological replicates with three technical replicates. Statistical significance was determined by Student’s *t*-tests. *: *P*-value <0.05. **(C)** Levels of H3K9me2 determined by ChIP-qPCR in WT, *edm2-2* and *edm2-3*. H3K9me2 levels were normalized to the total histone H3 levels. *ACTIN8* (ACT8) served as a control locus. Error bars represent SEM for two biological replicates with three technical replicates.

Besides *RPP4*, another member of the Col-0 *RPP5* NLR cluster AT4G16900 is also a direct target of HA-EDM2 (Fig 6A, S6 Table and S10 Fig). ChIP-qPCR experiments (Fig 6B) with *E2pro*:*HA-E2c* plants confirmed binding of HA-EDM2 to chromatin of the 3’ portion of this NLR gene and the Gypsy TE AT4TE42950 overlapping with its last four exons. While we did not observe a significant effect of EDM2 on the total transcript levels at AT4G16900, levels of H3K9me2 appear altered at this locus in *edm2* plants (Fig 6A). However, unlike the situation at *RPP7* and *RPP4*, H3K9me2 levels at AT4G16900 are slightly higher in *edm2* mutants compared to WT (Fig 6A and 6C, S1 Table). As we previously observed for the *RPP4*-associated TE *COPIA4*, H3K9me2 levels in the AT4G16900-associated TE, AT4TE42950, are elevated in *edm2* plants (Fig 6A and S2 Table). While the transcript levels over the entire length of this Gypsy transposon are not significantly changed, we observed in *edm2* a substantial reduction in transcript levels of its polyprotein encoding region (AT4G16910; Log2 fold transcript change = -6.098, adjusted *p*-value = 4.92E-05; Fig 6A). Thus, despite the fact that our experiments did not uncover a significant effect on transcripts in the TE-associated NLR gene AT4G16900, EDM2 has a local impact on H3K9me2 levels (affecting both the NLR gene and TE) and transcripts of the polyprotein-encoding region of this TE.

**Fig 7:**
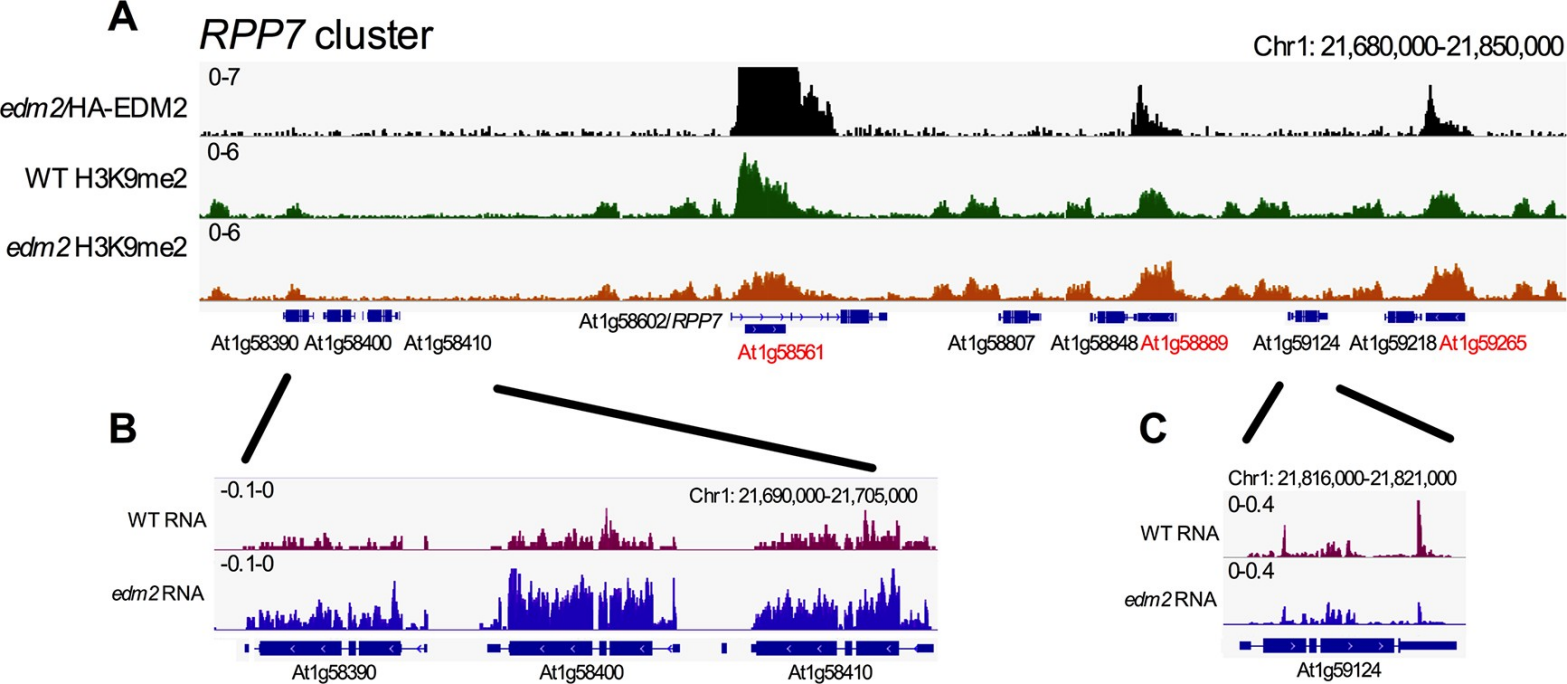
EDM2 affects members of the Col-0 *RPP7* NLR cluster. **(A)** Genome browser view of normalized HA-tagged EDM2 ChIP-seq and H3K9me2 ChIP-seq (WT and *edm2-2*) at the *RPP7* cluster. Eight NLR genes are labeled in black; three Copia-type TEs are labeled in red. **(B and C)** Genome browser view of normalized RNA-seq data for AT1G58390, AT1G58400, AT1G58410 **(B)** and AT1G59124 **(C)** of the *RPP7* cluster. The y*-*axis represents coverage values (normalized per million mapped reads).

Besides *RPP4*, AT4G16900 and *RPP7*, our genome profiling analysis suggested effects of EDM2 on H3K9me2- and/or transcript levels of 56 additional NLR genes (S4 Table). These include four additional members of the *RPP7* cluster (AT1G58390, AT1G58400, AT1G58410 and AT1G59124; Fig 7), two additional members of the Col-0 *RPP5* cluster (AT4G16920 and AT4G16960) and one member of *RPP1* cluster (AT3G44400) (S4B Table). Our HA-EDM2 ChIP-seq results showed HA-EDM2 associated with all three *RPP7* cluster-associated TEs. Besides *RPP7* that harbors *COPIA-R7*, the other two members of the eight NLR gene-comprising *RPP7* cluster associated with TEs are AT1G58848 and AT1G59218 (Fig 7A and S6 Table). In each of these two cases, binding of HA-EDM2 is centered on a TE (AT1G58889/AT1TE71950 and AT1G59265/AT1TE72060) immediately adjacent to the respective NLR. Additional examples of NLRs affected by EDM2 include AT3G44630 (another member of the *RPP1* gene cluster) and AT5G44870 (S11 Fig), for which we also found EDM2 to be associated with TE fragments (AT3TE65615 and AT5TE65325, S11 Fig) immediately adjacent to the respective NLRs. Neither AT4G16900, nor any of the additional NLRs described in this section as potentially EDM2-controlled (AT1G58390, AT1G58400, AT1G58410, AT1G59124, AT1G58848, AT1G59218, AT3G44400, AT3G44630, AT4G16920, AT4G16960 and AT5G44870) have been reported to be functional *R* alleles in Col-0 and are not known to mediate disease resistance against a pathogen. Hence, we could not test if *edm2* plants are compromised in the immune function of any of these NLRs.

### TEs are tightly associated with Arabidopsis NLR loci

This study demonstrates a broad role of EDM2 in controlling silencing marks at TEs. Over 2,000 TEs, predominantly Copia and Gypsy retrotransposons, exhibit altered H3K9me2 and/or transcript levels in *edm2-2* plants compared to WT. Our genome profiling analysis further uncovered effects of EDM2 on H3K9me2 and the expression of numerous Arabidopsis NLR genes. As for *RPP7*, the effect of EDM2 on these genes seems, at least in some cases, linked to roles of EDM2 in controlling silencing states of nearby TEs. TEs have long been suspected to be critically important for the fast evolution of structural NLR diversity by facilitating non-homologous recombination events in NLR gene clusters [39, 40]. Together with previous results [4, 18, 29–31] our new findings also suggest a broad role of TEs in equipping rapidly evolving NLR genes with regulatory mechanisms allowing for balanced and tightly controlled expression. Such roles in NLR evolution are likely not limited to TEs controlled by EDM2 and may generally apply to a wide range of active mobile elements. In this case natural selection should favor close associations between TEs or TE fragments and NLR genes. In order to examine if NLR loci generally tend to be tightly associated with TE DNA, we determined the genome-wide association between Arabidopsis NLR genes and TE location.

**Fig 8:**
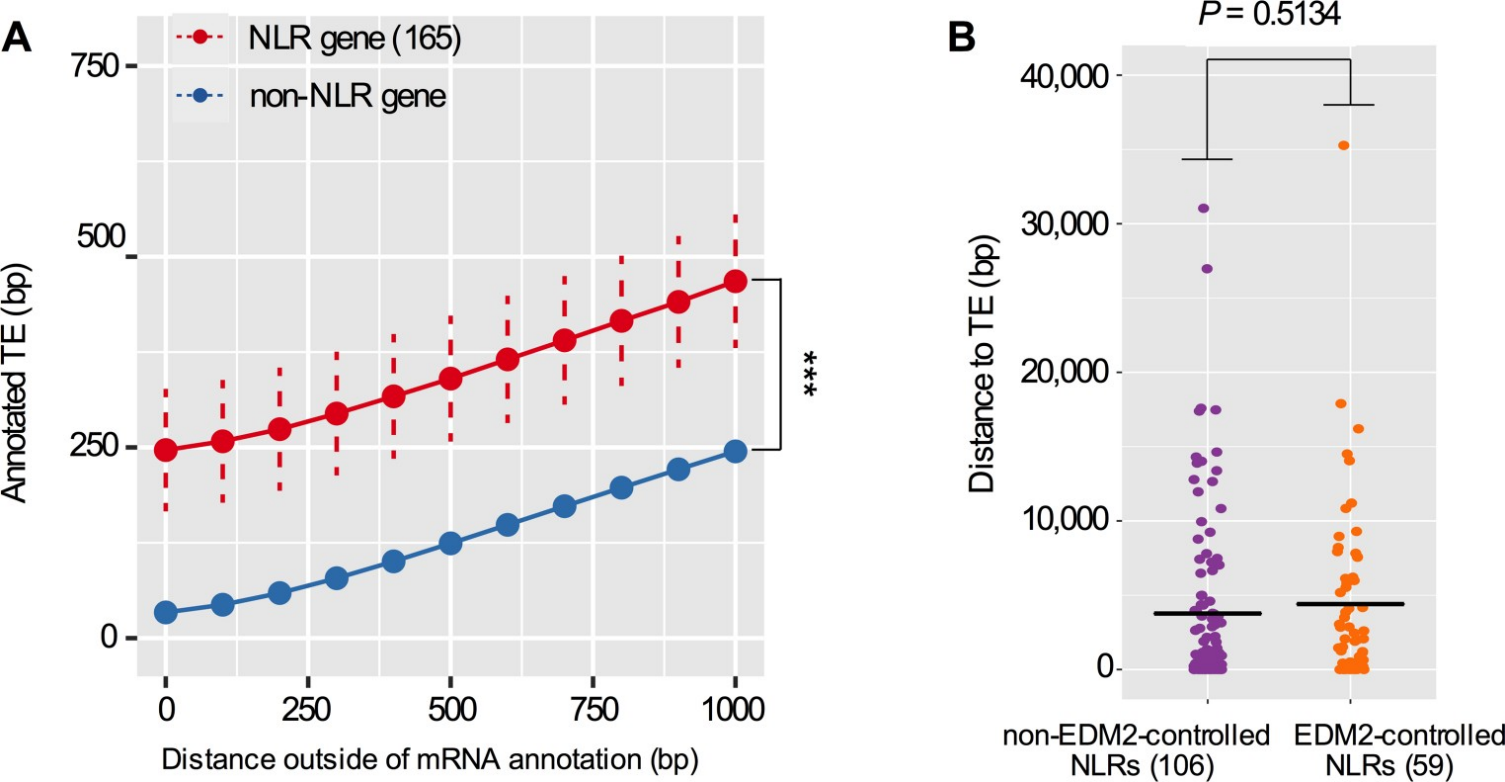
TEs closely associate with NLR genes in Arabidopsis. **(A)** Distances outside of the annotated mRNA gene space (from transcriptional start site to polyadenylation site) of NLR genes and non-NLR genes (x-axis) are plotted against the average number of annotated TE (TE) base pairs present in the respective area (y-axis) for each group of genes. Dashed bars represent 95% confidence intervals. Statistically significant differences between NLR and non-NLR genes have been calculated using ANOVA and are for all comparisons between NLR and non-NLR genes. ***: *P*-value = 5.775357e-29.**(B)** Average distance to the nearest TE for the 59 EDM2-controlled NLRs compared with the remaining 106 annotated NLRs in the Arabidopsis genome. The black bars represent 4,405.68 bp for 59 EDM2-controlled NLRs and 3,768.04 bp for the 106 NLR genes. An unpaired *t*-test showed that there is no significant difference between both examined gene sets.

We found within NLR gene space, NLR genes to have an average of 236 bp more annotated TE compared to non-NLR genes (x-axis = 0 in Fig 8A). We expanded our analysis up to 1,000 bp beyond the NLR gene space in both the upstream and downstream directions and found the same relationship: NLR genes are more closely associated with DNA annotated as TEs compared to non-NLR genes (x-axis = 1–1,000 in Fig 8A). Regarding the distance to the nearest TE, we did not observe a significant difference between the 59 EDM2-controlled NLRs and the rest of 106 NLRs in the Arabidopsis genome (Fig 8B), suggesting that the close association with TEs is a general feature of NLR genes, including those controlled by EDM2. Thus, tight association with TEs is not limited to EDM2-controlled NLRs and is broadly observed for these immune receptor genes, irrespective of their dependency on EDM2. Consistent with this, we also observed effects of H3K9me2 marking non EDM2-targeted TEs on the expression of nearby NLR genes. Examples for such cases are illustrated in S12 Fig, which shows that transcript levels of two pairs of TE-associated NLRs are dependent on H3K9me2 and changed in the H3K9me2-deficient *suvh456* mutant (S12 Fig).

### The histone demethylase IBM1 controls H3K9me2 levels at several direct EDM2 target loci

Previous work suggested that EDM2 indirectly affects 5mCHG levels in thousands of Arabidopsis genes by promoting proper expression of IBM1, a H3K9 demethylase, which is responsible for removing H3K9me2 and 5mCHG in genes associated with heterochromatin [41–43]. EDM2 was found to bind to a heterochromatic region within a long IBM1 intron and to promote the production of mRNAs encoding the full length IBM1 protein [16]. Our data confirm binding of HA-EDM2 to this region (S7B Fig). However, previous genome-profiling work [16] also showed EDM2 to have IBM1-independent functions, as differential CHG methylated regions have been observed that are not common to mutants of both *EDM2* and *IBM1*, respectively, and only affected in one of them. In order to examine the involvement of IBM1 in H3K9me2 regulation at loci targeted by EDM2, we performed H3K9me2 ChIP-seq with the *ibm1-4* mutant (SALK_035608C). We identified 6,082 genes and 2,706 TEs with significant changes in H3K9me2 levels in *ibm1-4* compared to WT (S7 and S8 Tables). While effects of IBM1 on H3K9me2 at TEs have not been reported before, its effects on this silencing mark in genes are consistent with previously published ChIP-chip data [44]. In our and previous studies [44] the majority of significant changes in *ibm1-4* are H3K9 hyper-dimethylation in genic regions. As anticipated, we observed a large overlap between the *ibm1-4* and *edm2-2* specific H3K9me2 profiles. This set of overlapping genes includes 11 NLRs (S4A and S4E Table). Unexpectedly, however, we observed an effect of *IBM1* on H3K9me2 levels in multiple genes and TEs, which are directly targeted by EDM2, including 18 out of 46 HA-EDM2-associated TEs and genes such as *RPP7*, and *EDM2* itself (Fig 2A and S7 Fig). In all these cases the effects of EDM2 and IBM1 are co-directional (S9 Table). For example, in both *ibm1-4* and *edm2-2* H3K9me2 levels are reduced at *RPP7* and increased in both mutants at *EDM2*. Thus, regulatory interactions between EDM2 and IBM1 seem to be more complex than initially anticipated. EDM2 appears not to suppress TE silencing marks in genic regions solely indirectly by promoting IBM1 expression. At least in some cases IBM1-affected loci are also directly targeted by EDM2, possibly suggesting cooperative interactions of both proteins in controlling H3K9me2 levels.

Feedback mechanisms may further be responsible for reciprocal effects of *IBM1* and *EDM2* on each other’s H3K9me2 status and both, EDM2 and IBM1, affect H3K9me2 at numerous TE-associated NLRs (including *RPP7* and the *RPP5* cluster members AT4G16900 and AT4G16920, Figs 2 and 6A; S4 Table). To gain further insight regarding regulatory interactions between *EDM2* and *IBM1*, we compared RNA-seq profiles in the *edm2-2* (from this study) and *ibm1-6* [45] mutants for those 51 NLRs (S4 Table) that are differentially expressed in *edm2-2*. As shown in supplementary figures (S13 to S18 Figs) various different patterns can be discriminated regarding the dependency of these NLRs on *EDM2* and *IBM1* and the direction of their respective transcript level changes. Some NLRs are jointly transcriptionally up-regulated in both *edm2* and *ibm1* plants, while in many other cases effects of *edm2* on NLR transcript levels are not reproduced or even reciprocal in *ibm1*. These observations further support that (1) *EDM2* has *IBM1* independent functions and (2) interactive relationships between *IBM1* and *EDM2* are complex and variable.

## Discussion

EDM2 was previously shown to be a chromatin-associated protein controlling expression of the RPP7 immune receptor and the histone H3K9 demethylase IBM1 by promoting the synthesis of full-length transcripts encoding these proteins. EDM2 has also been reported to control silencing states of some TEs, and its physical association with heterochromatic TE/repeat sequences was found to be critical for its function in *RPP7/IBM1* regulation. Through regulation of IBM1, EDM2 has also been implicated in global suppression of CHG methylation in genic regions associated with heterochromatin. In this study, we expanded on previous findings by genome-wide H3K9me2 ChIP-seq, RNA-seq and HA-EDM2 ChIP-seq analysis. Our new data show that EDM2 controls genome-wide expression of NLR genes and TEs, as we observed in *edm2-2* plants effects on H3K9me2 and/or transcript levels at 59 NLR genes and over 2,000 TEs.

In some cases, the involvement of EDM2 in NLR gene regulation resembles its role in controlling *RPP7* expression. EDM2 is physically associated with chromatin of the Columbia *RPP5* cluster members *RPP4* and AT4G16900 as well as TEs directly adjacent to these NLR genes. For both *RPP4* and AT4G16900, H3K9me2 seems to have spread from the adjacent TEs into genic regions of both NLRs. As with *RPP7*, EDM2 promotes the synthesis of full-length transcripts of *RPP4* and suppresses its proximal transcript polyadenylation/termination. This effect is also dependent on high H3K9me2 levels at the respective proximal polyadenylation site.

Effects of EDM2 on *RPP4* expression are complex, as we found it also to increase expression of the TIR domain-encoding exon 1 of this NLR. If this observation is related to the use of alternative transcription start-sites identified downstream from exon 1 [36] remains to be examined. Furthermore, EDM2 was previously found to promote the alternative splicing-associated generation of RPP4-COPIA4 fusion transcripts consisting of RPP4 exon 1 and a part of COPIA4, but lacking the remaining RPP4 exons [46, 47]. Consistent with its multi-facetted role in controlling *RPP4* expression, EDM2 is required for maximal function of *RPP4* in mediating resistance against the *H*. *arabidopsidis* isolate Emoy2 in Arabidopsis cotyledons. Highlighting the critical importance of *COPIA4* in this context, mutation of this retrotransposon by a T-DNA insertion or silencing of its expression resulted in reduced cotyledon resistance against another *RPP4*-recognized *Hpa* isolate, Emwa1 [46].

While EDM2 promotes expression of a small number of NLR genes (11 NLR genes including *RPP7*, *RPP4* and At4g16900), it acts as a suppressor of a significantly larger set of NLR genes (40 NLR genes, Fig 3B). None of these EDM2-suppressed NLR genes is associated in our ChIP-seq study with EDM2-HA. Thus, contrary to *RPP7*, *RPP4* and At4g16900, these 40 NLR genes are unlikely direct targets of EDM2. We also did not observe any common effect of EDM2 on H3K9me2 levels within the entire set of 40 suppressed NLR genes. Nor do these genes share associations with TEs (see below). Thus, mechanistic details, of the suppressive effect of EDM2 on these 40 NLR genes may vary from case to case and they are likely indirect targets of EDM2.

However, the observation that such a large number of NLR genes is suppressed by EDM2, while the sets of all genes it acts on as a positive and negative regulator are equally large, appears compelling to us. Clearly, EDM2 has evolved into a role as a broad suppressor of NLR genes in Arabidopsis. Such a role is consistent with the enhanced defense against *Hpa*Noco2 and *Pseudomonas syringae* as well as reduced fitness we observed in *edm2* plants, in which (compared to WT) almost four-times more NLR genes are up-regulated than down-regulated and which, therefore, likely exhibit a strong net-increase of NLR background activity. Up-regulation of NLR expression has been linked to reduced fitness and constitutive immunity in several Arabidopsis mutants before. For example, the *bal* mutant contains an extra copy of the NLR gene *SNC1* and, consequently, exhibits elevated expression of this gene as well as substantially stunted growth, activated defense response mediated by salicylic acid and enhanced immunity against *Pseudomonas syringae* bacteria [7, 8]. The *cpr1* mutant is deficient in an F-box protein mediating degradation of the NLRs SNC1 and RPS2. Higher levels of these NLRs are correlated with reduced growth and enhanced immunity against various pathogens in this mutant [48, 49]. Nonetheless, we cannot exclude that enhanced NLR expression in *edm2-2* is a consequence of some disturbance of cellular homeostasis associated with reduced fitness and an indirect pleiotropic effect, rather than the cause of fitness costs. However, we consider this as unlikely, given that causality between NLR overexpression and reduced fitness has been established in several cases [7, 8, 48, 49].

Balancing the needs for sufficient expression of NLR immune receptors with preventing fitness penalties arising from their background activities requires sophisticated solutions for genome-wide NLR expression control. In this important function, EDM2 seems assisted by its interaction partner, the RNA binding protein EDM3. Re-analyzing transcript profiles obtained with the *EDM3* mutant *aipp1* [19], we observed very similar effects on NLR genes. While largely equal numbers of genes are up- or down-regulated in this mutant, its ratio between up-and down-regulated NLRs is even more distorted and shifted towards up-regulated NLRs than in *edm2* (S19 Fig). Fifty percent of all NLRs up-regulated in *edm2* are also up-regulated in *aipp1* (S20A Fig). Thus, EDM2 and EDM3 have very similar roles in global NLR expression control and may cooperate to a large degree in this function.

The EDM2 and EDM3 target gene *IBM1* also affects expression of large numbers of NLR genes (S20 Fig). However the numbers of NLR genes up- or down-regulated by this histone demethylase are equally large (S19 and S20 Figs) and only a small number of NLR genes up-regulated in *ibm1* mutant plants are also up-regulated in the *edm2* and *aipp1* mutants (S20A Fig). Thus, IBM1 is unlikely to contribute to the specific role in NLR gene suppression that EDM2 and EDM3 have evolved into and its effect on NLR genes is rather a consequence of its general function as a genome-wide operating H3K9 demethylase. Consistent with this view is that unlike *edm2* mutants, which are more resistant to the *Pseudomonas syringae* DC3000 strain, *ibm1* mutants show the opposite phenotype and behave more susceptible to these virulent bacteria [50].

In addition to its role in controlling NLR gene expression we found EDM2 to control silencing states of a large number of transposons. Our results further show that the influence of EDM2 on the expression of some NLR genes seems recruited into the context of these genes by insertions of TEs. A large body of literature supports a critical function of TEs in the evolution of structural and functional diversity of plant NLR genes. NLR genes are one of the fastest evolving and structurally diverse gene families in plants. Their association with TEs and organization in complex gene clusters is believed to be critical for the fast pace of their structural and functional diversification [39, 51]. Frequent recombination events and other mutagenic processes at such loci followed by diversifying selection are believed to rapidly generate the structural diversity needed to match high AVR effector evolution rates in the microbial world [2, 40]. Close association of TEs with NLRs, and high enrichment of TEs in NLR clusters is often observed [52–54]. For example, both the Arabidopsis *RPP5* and *RPP7* clusters in the Col-0 accession contain eight closely related NLR genes and three annotated transposons [55, 56]. Taken together these observations strongly suggest that TEs can play important roles contributing to the fast pace of NLR evolution. Consistent with this view, we observed a statistically highly significant association of TEs with NLR loci in Arabidopsis. Our results on EDM2 strongly imply that, besides serving as a major driver of structural NLR diversification, TEs have a second important role in NLR evolution, by providing the raw material for gene regulatory mechanisms. It is well documented that TE insertions can recruit *cis*-regulatory sequences as well as epigenetic features into the context of genes [29–31, 57, 58]. Our previous [18] and current studies provided evidence for both. We found 5’LTR sequences of the *COPIA-R7* and *COPIA4* retrotransposons to serve as polyadenylation signals at *RPP7* and *RPP4*, respectively. In both cases (and in the case of AT4G16900), transposons also seem to nucleate the formation of H3K9me2, which spreads into the neighboring genes affecting their expression. Interestingly, this heterochromatic transposon silencing signal does not silence the genes’ transcription (promoter silencing), but serve the different function to suppress proximal genic transcript polyadenylation. As we observed for *RPP7*, *RPP4*, AT4G16900, AT1G11270 and AT3G05410, EDM2-mediated effects on H3K9me2 and/or transcript levels are likely recruited by insertions of TE insertions to these sites.

Besides *COPIA-R7*, additional TE sequences affect the complex regulation of *RPP7*. Several TEs and other repeat sequences in its 5’UTR seem to attract massive cytosine methylation to a region directly upstream of the transcription start-site [59]. However, cytosine methylation in this region is suppressed by demethylases to a level that allows for sufficient levels of transcription of this gene. Furthermore, we found the *SimpleHAT* DNA transposon in the distal promoter of *RPP7* to provide a docking site for an unknown DNA binding activity and to serve as a pathogen-responsive *cis*-element [4]. Additional examples (besides *COPIA-R7* and *COPIA4*) illustrating the ability of TEs to equip NLR genes with regulatory mechanisms include the *AtCOPIA93*-derived solo LTR upstream from *RPP4*, which mediates pathogen-responsive transcriptional upregulation of this NLR gene [58], the LTR retrotransposon *Renovator* which is inserted upstream of the rice NLR gene *Pit* and provides promoter sequences directing high transcript levels of this gene [31], as well as the tobacco NLR gene *N*, which contains the miniature inverted-repeat transposable element *MiS1-1*, that serves as a differentially expressed alterative exon of critical importance for the function of this disease resistance gene [29, 30].

While recruitment of regulatory mechanisms by TEs is observed for many types of genes [57], it is likely of particular importance in the case of NLRs. Firstly, as outlined above, homeostasis of NLR gene expression is critically important. Thus, NLR genes need to be equipped with a set of mechanisms tightly controlling their base expression levels and at the same time allowing for transient expression changes if circumstances require this, such as during defense induction [10]. Secondly, NLR evolution has to progress at an unusually fast pace, to match high evolution rates of AVR effectors in the microbial world. TEs as potential mutagens and catalysts of recombination as well as distributers of regulatory mechanisms are likely uniquely suited to promote fast NLR evolution by affecting both structure and expression of these genes. Of particular benefit for plants in this respect may be the fact that TE expression and mobilization is often inducible by pathogen infections and other stress-related stimuli [60, 61]. At least in Arabidopsis, this seems to be partially due to a transient reduction of global 5mC and H3K9me2 levels in response to defense induction [18, 62].

While our study provides examples related to EDM2-controlled NLRs, the general statistical association of TEs with NLR genes suggests that this role extends to a wide variety of different TEs. From a broader perspective, TEs are also emerging as important factors for the function and evolution of pathogen effector genes and proteins that promote virulence or are recognized as avirulence proteins by NLR receptors [63, 64]. Thus, gene-for-gene co-evolution is likely driven by similar factors in plant and pathogen genomes.

The dual nature of EDM2 functions in NLR expression control (promoting expression of some, while suppressing others) is unlikely coincidental and both functions may be causally linked. Numerous studies have implied that plants have limited tolerance for NLR expression, and that certain thresholds levels for NLR transcripts cannot be exceeded [3, 4, 65, 66]. For example, a phasi-RNA-based control system inherent to several plant species appears to restrict total levels of NLR transcripts to certain limits. Interesting in this context is that *RPP7*, which requires EDM2 for optimal levels of expression, causes substantial loss of fitness in Arabidopsis. Loss-of-function mutants of *RPP7* are clearly more vigorous than wild type plants (Lai et al. manuscript in preparation). Based on these observations, it is tempting to speculate that the role of EDM2 in global NLR gene suppression serves the purpose to compensate for fitness penalties caused by high expression of *RPP7* and possibly other NLRs, whose expression is promoted by EDM2. Cooption of EDM2 to roles in promoting expression of certain NLRs may not have been possible without suppressing at the same time expression of other members of this immune receptor gene family.

While our results provided substantial new insight into genome-wide roles of EDM2, they leave room for speculation regarding EDM2’s mechanisms of action at its target genes. We previously observed that gene regulatory roles of EDM2 are context dependent and vary from locus to locus [21, 22]. Our ChIP-seq experiments uncovered significant levels of association of HA-EDM2 with only 78 loci. The EDM2 protein seems quite unstable only accumulating to very low levels in cells (HA-EDM2 in *E2pro*:*HA-E2c* plants is undetectable by Western Blotting). Thus, *in vivo*-detection of this protein by ChIP appears only possible at a limited set of strong target sites. While our ChIP-seq experiments allowed us to clearly establish certain direct target sites of EDM2, we have almost certainly missed many loci bound by this protein, limiting our ability to draw conclusions about its general mechanistic roles in gene regulation. Only at a small number of loci, such as *RPP7* and *IBM1*, such mechanistic details have been uncovered. In each of these cases, EDM2 serves as a chromatin binding protein, recruiting the RNA binding proteins ASI1/IBM2 and EDM3/AIPP1 to intronic heterochromatic regions [16, 18–20, 67, 68]. Together with additional factors, such as AIPP2, AIPP3 and AIPP4 [19] the EDM2-containing complex controls alternative polyadenylation. While at *RPP7* and *IBM1*, this complex promotes the synthesis of full-length transcripts, by suppressing proximal polyadenylation, the outcome at other loci may be different and affected by differences of the stoichiometry and function of the various complex components.

Most effects observed in our study are likely indirect and mediated secondarily by EDM2 controlled regulators. Some of these are mediated via the H3K9-demethylase IBM1 [16]. Our observation that EDM2 enhances transcript levels of 323 genes by suppressing H3K9me2 (Fig 1E) is consistent with the role of EDM2 in promoting proper IBM1 expression. Besides *IBM1*, we also found 299 transcription factor genes to be affected by EDM2. These regulators likely cause additional indirect effects and may be responsible for many EDM2-dependent transcript level changes not associated with changes of H3K9me2 levels.

Like all other types of genes differentially expressed in *edm2-2*, EDM2-controlled NLR genes vary regarding changes of H3K9me2 and their dependency on IBM1 (S13–S18 Figs). Only three of them are directly bound by HA-EDM2 and only some of them are associated with EDM2-regulated TEs or affected by EDM2-mediated alternative polyadenylation. Thus, EDM2 appears to execute its important function of balancing NLR gene expression via multiple disparate processes that likely involve additional regulatory factors. Future studies will have to address mechanistic details underlying their coordinated roles and functional connections to EDM2 in NLR gene regulation.

## Methods

### Plant material and growth conditions

The Arabidopsis ecotype Columbia (Col-0) and *ibm1-4* (SALK_035608C) were obtained from the Arabidopsis Biological Resource Center (ABRC, Ohio State University). The *edm2-2* (SALK_014520), *edm2-3* (SALK_114312) [14], transgenic complementation lines (*E2pro*:*HA-E2c*) [21] and Oy1 [34] were described previously. The Col-0 *suvh4 suvh5 suvh6* triple mutant (*suvh456*) was kindly provided by Dr. Judith Bender (Brown University, Providence, RI). All Arabidopsis mutants used in this study are in Col-0 background. All Arabidopsis seedlings were grown on soil or half-strength Murashige and Skoog (1/2 MS) solid medium containing 1% (w/v) sucrose in a growth chamber (16-h day, 8-h night, 22°C; 100 μE m^-2^s^-1^).

### RNA isolation and qRT-PCR analysis

Aerial parts of 2-week-old plants were harvested from 1/2 MS solid plates and used for total RNAs isolation. Total RNA was isolated using TRIzol reagent (Life Technologies, Invitrogen) and treated with TURBO DNA-free^TM^ kit (Ambion, Life Technology, Invitrogen). Reverse transcription was conducted by Maxima reverse transcriptase (Thermo Fisher Scientific, Waltham, MA, USA) with 100 pmol of oligo (dT)_18_. qRT-PCR was performed with the CFX Connect detection system (Bio-Rad) using iQ SYBR Green Supermix (Bio-Rad, Hercules, CA, USA). Actin8 was served as an internal control. All the primers used for qRT-PCR are listed in S10 Table.

### *H*. *arabidopsidis* inoculation

The growth condition, propagation and application of *H*. *arabidopsidis* isolate *Hpa*-Emoy2 were described previously [69]. Two-week-old seedlings were spray-inoculated with spore suspensions (3–5 × 10^4^ spores/mL) using Preval sprayers (Preval, Coal City, IL, USA). The extent of infections was determined by counting visual sporangiophores at 7 dpi.

### RACE

The 3’ RACE were carried out using the GeneRacer^TM^ kit (Life Technologies, Invitrogen), following the manufacturer’s instruction. All primers used for RACE are listed in S10 Table.

### Chromatin immunoprecipitation

Aerial parts of 2-week-old plants were harvested from 1/2 MS solid plates and two grams of seedlings per sample were used for Chromatin Immunoprecipitation (ChIP) assays. ChIP was performed as descried previously [18] using anti-H3K9me2 (ab1220, Abcam, Cambridge, MA, USA), anti-H3 C-terminal (61277, Active Motif, Carlsbad, CA, USA) and anti-HA (AB9110, Abcam, Cambridge, MA, USA) antibodies. All the antibodies are commercially available and were previously successfully used in Arabidopsis [18, 43]. All primers used for ChIP-qPCR are listed in S10 Table.

### H3K9me2 ChIP-seq and data analysis

ChIP-seq libraries were prepared using NEBNext Ultra^TM^ II DNA library prep kit for Illumina (E7645, New England Biolabs) according to the manufacturer’s instruction. Libraries were sequenced on the Illumina NextSeq500 generating 75 bp -single-end sequence reads.

For each Chip-seq library, raw reads quality was first analyzed using FastQC (https://www.bioinformatics.babraham.ac.uk/projects/fastqc/), and any base with a quality score below 25 or N was trimmed using Sickle (https://github.com/najoshi/sickle). Trimmed reads were then mapped to the *A*. *thaliana* genome (TAIR 10) using BWA 0.7.15-r1140 with mem option default parameters [70]. See S11 Table for read statistics. Uniquely mapped reads were further filtered for calculating H3K9me2 coverage in transcripts, while unfiltered reads were used for calculating H3K9me2 coverage in transposable elements. The number of reads mapped to each transcript was determined using BEDTools v2.25.0 [71], and Spearman correlation coefficients were calculated between biological replicates. To compare H3K9me2 level between WT and *edm2-2* samples, non-expressed transcripts with coverage value below 1 in all libraries were removed. Transcripts representing differentially methylated regions were determined using DEseq2 in R [72] with a *P*-adjusted value of 0.05 and a 1.2 fold change.

### RNA-seq and data analysis

Total RNAs were isolated from two-week-old plants grown in 1/2 MS solid plates using TRIzol reagent (Life Technologies, Invitrogen). Ten micrograms of total RNA were treated with TURBO DNA-free^TM^ kit (Ambion, Life Technology, Invitrogen) to eliminate the genomic DNA contamination. Five micrograms of DNA-free total RNA was treated with Ribo-Zero rRNA Removal Reagents (Plant Leaf)(Epicentre, USA) to remove Ribosomal RNA. RNA-seq libraries were prepared using NEBNext Ultra^TM^ Directional RNA library prep kit for Illumina (E7420, New England Biolabs) according to the manufacturer’s instructions. Libraries were sequenced on the Illumina NextSeq500 platform generating 2×75 bp pair-end sequence reads.

After sequencing, the quality of raw reads was analyzed using FastQC (https://www.bioinformatics.babraham.ac.uk/projects/fastqc/). The first 10 bases and the last base were trimmed. Contaminating adaptor reads, reads that were unpaired, bases below 25 and Ns, and reads shorter than 18 bases are also filtered using Sickle (https://github.com/najoshi/sickle). All trimmed reads were mapped to *A*. *thaliana* genome (TAIR 10) using HISAT2 v2.1.0 [73] with a known splice site file, built from Araport annotation file v11, and strand information parameter,—rna-strandness RF. See S11 Table for mapping statistics. Uniquely mapped reads were further filtered for calculating coverage in transcripts, while unfiltered reads were used for calculating coverage in transposable elements. Coverage values for each transcript were calculated using BEDTools with–split, -D, and -S parameters [71]. Spearman correlation coefficients between biological replicates were calculated and differentially expressed transcripts were determined using DEseq2 [72] with a *P*-adjusted value of 0.05 and a 1.2 fold change. RNA-seq raw data for *ibm1-6* and the corresponding WT were obtained from NCBI (GEO: GSE93024) and processed using the same parameters described above.

### Genome browser tracks

For genome browser tracks, read coverage per nucleotide is calculated using BEDTools. Coverage values were then normalized per million mapped reads. Genome tracks were displayed in IGV (Integrative Genome Viewer) [74]. For HA-tagged EDM2 samples, signals from biological replicates were first combined, and background signal measured by input sample analysis was subtracted from *edm2-2* and WT samples. To visualize HA-EDM2 associated areas, signals from WT were further subtracted from *edm2-2* signals. Genome browser tracks for *suvh456* RNA-seq data were obtained from NCBI (GEO: GSE111609) [75].

### Statistical analysis of TE distribution and TE NLR associations

The proportion of TE distribution was calculated based on the ratio of TE family size divided by the total size of TEs present in each category. Significant differences between the proportion of EDM2-controlled TEs and the proportion of TEs within the chromosome arms or pericentromeric regions was determined using annotation from Kawabe et al. [76] and the Fisher exact test. In order to correctly use the Fisher exact test, we randomly distributed an equal number of TEs 1,000 times within either the chromosome arms or pericentromeric regions and reported the average *P*-value for all re-sampling in Fig 4D and 4E. 165 NLR genes and non- NLR genes (28,610) are from the Arabidopsis TAIR10 annotation. Using *bedmap*, we calculated the cumulative length of TE annotation (from TAIR10) present in the surrounding area of the genic mRNA from 0 to 1,000 bp with a step of 100 bp. Statistical significance was tested using an ANOVA test by randomly re-sampling 1,000 time 165 non- NLR genes and reported the average *P*-value.

### *Pseudomonas syringae* pv. *tomato* DC3000 (*Pst* DC3000) inoculation

Bacterial growth and inoculation were performed as previously described [77]. Two-week-old plants were sprayed with *Pst* DC3000 suspension containing 2 × 10^8^ cfu/mL in 10 mM MgCl_2_ with 0.04% silwet L-77. Leaves were harvested at 3 hrs, 2 dpi and 3 dpi. Each leaves sample was weighed, surface sterilized in 70% ethanol for 30 s and washed in sterile distilled water for 30 s before ground in 10 mM MgCl_2_. Serial dilutions were plated on King’s B plates with appropriate antibiotics. Each data point represents the average of three replicates. Each replicate contains the bacterial titer of average of three individual pots with a density of 20 seedlings.

### Proteomics analyses of EDM2

Anti-HA beads were used to pull-down HA-EDM2 fusion protein after chromatin precipitation. The protein samples were trypsin-digested and analyzed with a MudPIT method described previously [78] with some modifications. MS1 scan range was m/z 400 to 1,400, and charge state was 2–6. For the MS2 scan, Orbitrap resolution was set at 15,000. A top speed mode with 2-sec and least-intense was used for both CID and HCD scanning. The decision-tree was targeted to a specific m/z in its inclusion list. The raw MS files were processed and analyzed using Proteome Discoverer v2.2 (ThermoFisher Scientific). Both Sequest HT and Mascot search engines were used to match all MS data to the Arabidopsis TAIR10 protein database supplemented with common contaminant proteins such as keratins.

## Supporting information

S1 Fig Genome browser view of normalized ChIP-seq signals for a representative section of Chromosome 1 (Chr1: 15, 600 kb -18, 000 kb). H3K9me2, H3C and input ChIP-seq for WT and *edm2-2* are shown in each tracks. The y*-*axis represents coverage values (normalized per million mapped reads).
(TIF)

S2 Fig Spearman correlation for replicates of ChIP-seq (A) and RNA-seq (B) analyses.
(TIF)

S3 Fig Significantly enriched GO (Gene Ontology) terms with *P* < 0.05 for *edm2-2* H3K9 hyper-dimethylated and transcriptionally down-regulated genes. (TIF)

S4 Fig Genome browser view of HA-tagged EDM2 ChIP-seq, H3K9me2 ChIP-seq and RNA-seq data at *RPP7* locus. The y*-*axis represents coverage values (normalized per million mapped reads).
(TIF)

S5 Fig **(A) Genome browser view of HA-tagged EDM2 ChIP-seq, H3K9me2 ChIP-seq and RNA-seq data at *RPP4* locus**. The y*-*axis represents coverage values (normalized per million mapped reads). **(B)** Genome browser view of WT and *ibm1* RNA-seq data at *RPP4* locus with different data ranges.
(TIF)

S6 Fig An LC/MS/MS spectrum is shown for a peptide ion with *m/z* 912.4314, 2+. MS2 fragmentation was achieved with HCD (higher-energy collision-induced dissociation) activation. All detected y-series as well as b-series fragment ions are labeled. Many neutral-loss fragments are also detected but not labeled here. Both Sequest HT and Mascot search engines matched this spectrum to an EDM2 peptide, 395—EISFEDIEDEDILTR—409, with high confidence. Summary for two mass spectrometry identified EDM2 peptides are shown in the bottom.
(TIF)

S7 Fig **(A and B) Genome browser view of HA-tagged EDM2 ChIP-seq, H3K9me2 ChIP-seq and RNA-seq data at the *EDM2* (A) and *IBM1* (B) loci.** The y*-*axis represents coverage values (normalized per million mapped reads).
(TIF)

S8 Fig **(A and B) Genome browser view of HA-tagged EDM2 ChIP-seq, H3K9me2 ChIP-seq and RNA-seq data at AT1G11270 (A) and AT3G05410 (B) loci.** The y*-*axis represents coverage values (normalized per million mapped reads).
(TIF)

S9 Fig Alternative polyadenylation sites at *RPP4* locus. **(A)** Schematic representation of *RPP4* with two alternative RNA transcript isoforms. **(B)** Nucleotide sequence of *RPP4* exon 6. Coding nucleotides of exon 6 are in upper case black letters. Lower case letters in red are 3’UTR. The 130 bp of 5’LTR sequence for *COPIA4* are shown in bold. Polyadenylation sites determined by 3’RACE are labeled by numbers 1–8. Blue underlined sequences between areas a (#5- #6) and b (#6- #8) indicate regions used for read counts shown in Fig 5B. **(C)** Amino acid sequence of protein isoforms encoded by *RPP4*. Purple: TIR domain; Blue: NB-ARC domain; Green: Leucine-rich repeat (LRR) domain, each one of 18 putative LRRs (predicted by Uniprot: F4JNA9) is underlined in pink. **(D)** Genome browser view of *edm2*, *suvh456* and respective WT RNA-seq data at *RPP4* 3’UTR. Red arrows indicate polyadenylation sites 5–8 shown in (B). Blue underlined areas a (#5- #6) and b (#6- #8) indicate regions used for read counts shown in Fig 5B. Genome tracks of three biological replicates were overlaid and displayed in IGV. Each single replicate is represented by the lightest shade. Overlaps between two replicates are of medium darkness, while overlaps of all three replicates are of maximal darkness. **(E)** Genome browser view of TSSs at *RPP4*. Genome tracks of two biological replicates for Col-0 were shown. TSS-seq data were obtained from NCBI (GEO: GSE113677)[36]. **(F and G)** Genome browser views of *edm2* and WT RNA-seq data at *RPP2A* (F) and *RPP2B* (G).
(TIF)

S10 Fig Genome browser view of HA-tagged EDM2 ChIP-seq, H3K9me2 ChIP-seq and RNA-seq data at AT4G16900. The y*-*axis represents coverage values (normalized per million mapped reads).
(TIF)

S11 Fig **Genome browser view of HA-tagged EDM2 ChIP-seq, H3K9me2 ChIP-seq and RNA-seq at AT3G44630 and AT3TE65615 (A) and AT5G44870 and AT5TE65325 (B) loci.** The y*-*axis represents coverage values (normalized per million mapped reads).
(TIF)

S12 Fig **Genome browser view of AT5G41740 and AT5G41750 (A) and AT5G47260 and AT5G47280 (B).** The y*-*axis represents coverage values (normalized per million mapped reads).
(TIF)

S13 Fig Genome browser view of H3K9me2 ChIP-seq and RNA-seq data at NLR loci significantly transcriptionally up-regulated in edm2 and ibm1. The AGI numbers for these loci are AT1G17600, AT1G17610, AT1G58390, AT3G07040, AT3G25510, AT3G44400, AT5G18360, AT5G46260, AT5G46520 and AT5G66630. The y-axis represents coverage values (normalized per million mapped reads).
(TIF)

S14 Fig Genome browser view of H3K9me2 ChIP-seq and RNA-seq data at NLR loci significantly transcriptionally up-regulated in *edm2* and showing no change in *ibm1*. The AGI numbers for these loci are AT1G12290, AT1G33560, AT1G56510, AT1G56540, AT1G63860, AT1G63880, AT1G72860, AT1G72870, AT3G50950, AT3G51560, AT3G51570, AT4G16960, AT4G27220, AT5G05400, AT5G17680, AT5G18370, AT5G22690, AT5G38340, AT5G41550, AT5G45240, AT5G46450, AT5G46510 and AT5G48770. The y*-*axis represents coverage values (normalized per million mapped reads).
(TIF)

S15 Fig Genome browser view of H3K9me2 ChIP-seq and RNA-seq data at NLR loci significantly transcriptionally up-regulated in *edm2* and down-regulated in *ibm1*. The AGI numbers for these loci are AT1G58400, AT1G63750, AT4G14370, AT4G33300 and AT5G58120. The y*-*axis represents coverage values (normalized per million mapped reads).
(TIF)

S16 Fig Genome browser view of H3K9me2 ChIP-seq and RNA-seq data at NLR loci significantly transcriptionally down-regulated in *edm2* and *ibm1*. The AGI numbers for these loci are AT3G14460 and AT3G14470. The y*-*axis represents coverage values (normalized per million mapped reads).
(TIF)

S17 Fig Genome browser view of H3K9me2 ChIP-seq and RNA-seq data at NLR loci significantly transcriptionally down-regulated in *edm2* and showing no change in *ibm1*. The AGI numbers for these loci are AT1G59124 and AT4G16920. The y*-*axis represents coverage values (normalized per million mapped reads).
(TIF)

S18 Fig Genome browser view of H3K9me2 ChIP-seq and RNA-seq data at NLR loci significantly transcriptionally down-regulated in *edm2* and up-regulated in *ibm1*. The AGI numbers for these loci are AT1G58602, AT1G59620, AT1G62630, AT1G72910, AT2G14080, AT4G16860 and AT5G43740. The y*-*axis represents coverage values (normalized per million mapped reads).
(TIF)

S19 Fig Distribution of EDM2, AIPP1 and IBM1-affected genes and NLRs that show significant up or down-regulated transcript level changes. *χ*^2^ test of independence showed significant differences between actual and expected equal distribution (50% up-regulated and 50% down-regulated genes). *: *P*-value <0.05. ***: *P*-value <0.001.
(TIF)

S20 Fig Venn diagram showing overlaps between *edm2-2*, *aipp1* and *ibm1-6* transcriptionally up-regulated (A) and down-regulated (B) NLR genes.
(TIF)

S1 Table List of genes that show significant H3K9me2 changes in *edm2-2* identified by ChIP-seq. (XLSX)

S2 Table List of TEs that show significant H3K9me2 changes in *edm2-2* identified by ChIP-seq. (XLSX)

S3 Table List of differentially expressed genes in *edm2* identified by RNA-seq. (XLSX)

S4 Table Arabidopsis NLRs show significant changes of H3K9me2 levels **(A)** and transcript levels **(B)** in *edm2*. **(C)** Arabidopsis defense-associated genes showing up-regulated changes of transcript levels in *edm2*. **(D)** Arabidopsis defense-associated genes showing down-regulated changes of transcript levels in *edm2*. **(E)** Arabidopsis NLRs show significant changes of H3K9me2 levels in *ibm1*.
(XLSX)

S5 Table List of differentially expressed TEs in *edm2* identified by RNA-seq. (XLSX)

S6 Table List of genes **(A)** and TEs **(B)** showing significant association with HA-EDM2 identified by mapping unique **(A)** or non-unique **(B)** reads.
(XLSX)

S7 Table List of genes that show significant H3K9me2 changes in *ibm1-4* identified by ChIP-seq. (XLSX)

S8 Table List of TEs that show significant H3K9me2 changes in *ibm1-4* identified by ChIP-seq. (XLSX)

S9 Table List of HA-EDM2 associated genes **(A)** and TEs **(B)** showing significant changes of H3K9me2 and/or transcript levels in *edm2* and/or *ibm1*.
(XLSX)

S10 Table Primers used in this study. (XLSX)

S11 Table Read mapping statistics for H3K9me2 ChIP-seq, HA-EDM2 ChIP-seq and RNA-seq. (XLSX)
