# Supplementary figures and images for: The Arabidopsis PHD-finger protein EDM2 has multiple roles in balancing NLR immune receptor gene expression
Source: PLoS Genet. 2020 Sep 14;16(9):e1008993. doi: 10.1371/journal.pgen.1008993 (PMC7529245; doi:10.1371/journal.pgen.1008993)

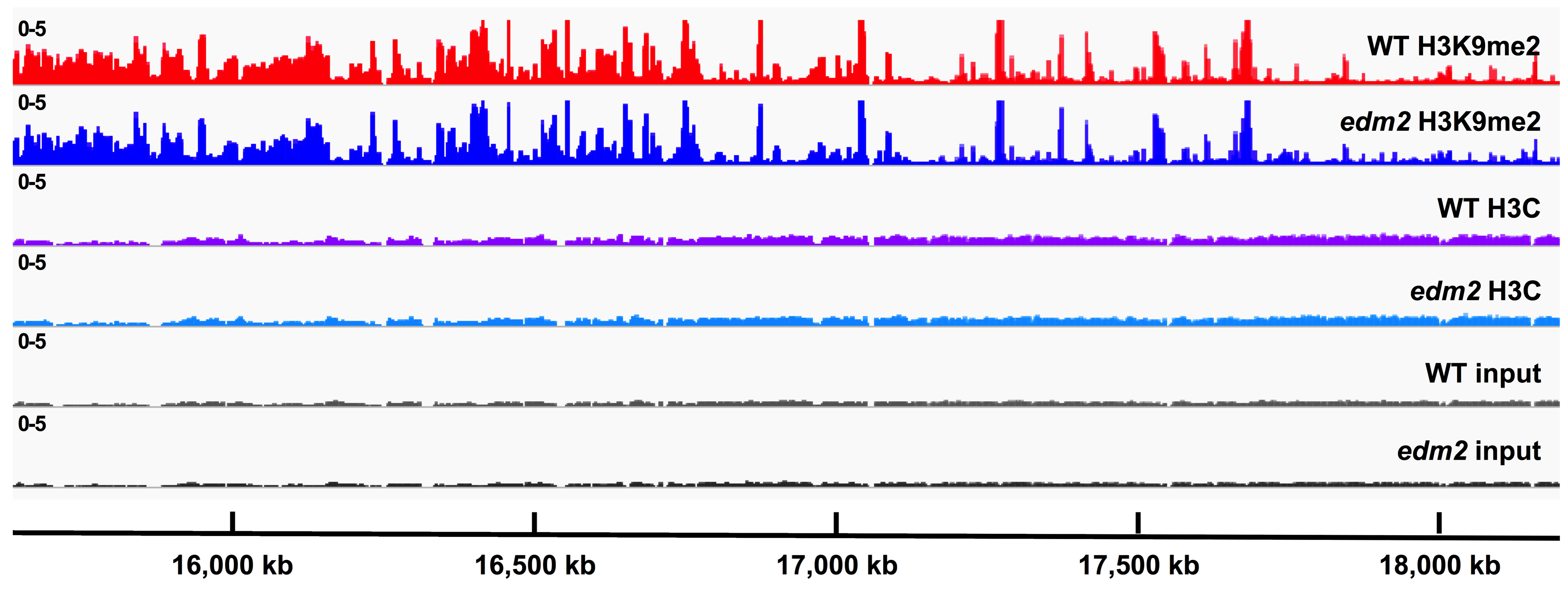

Supplement: S1 Fig — H3K9me2, H3C and input ChIP-seq for WT and edm2-2 are shown in each tracks. The y-axis represents coverage values (normalized per million mapped reads). (TIF) [file pgen.1008993.s001.tif]

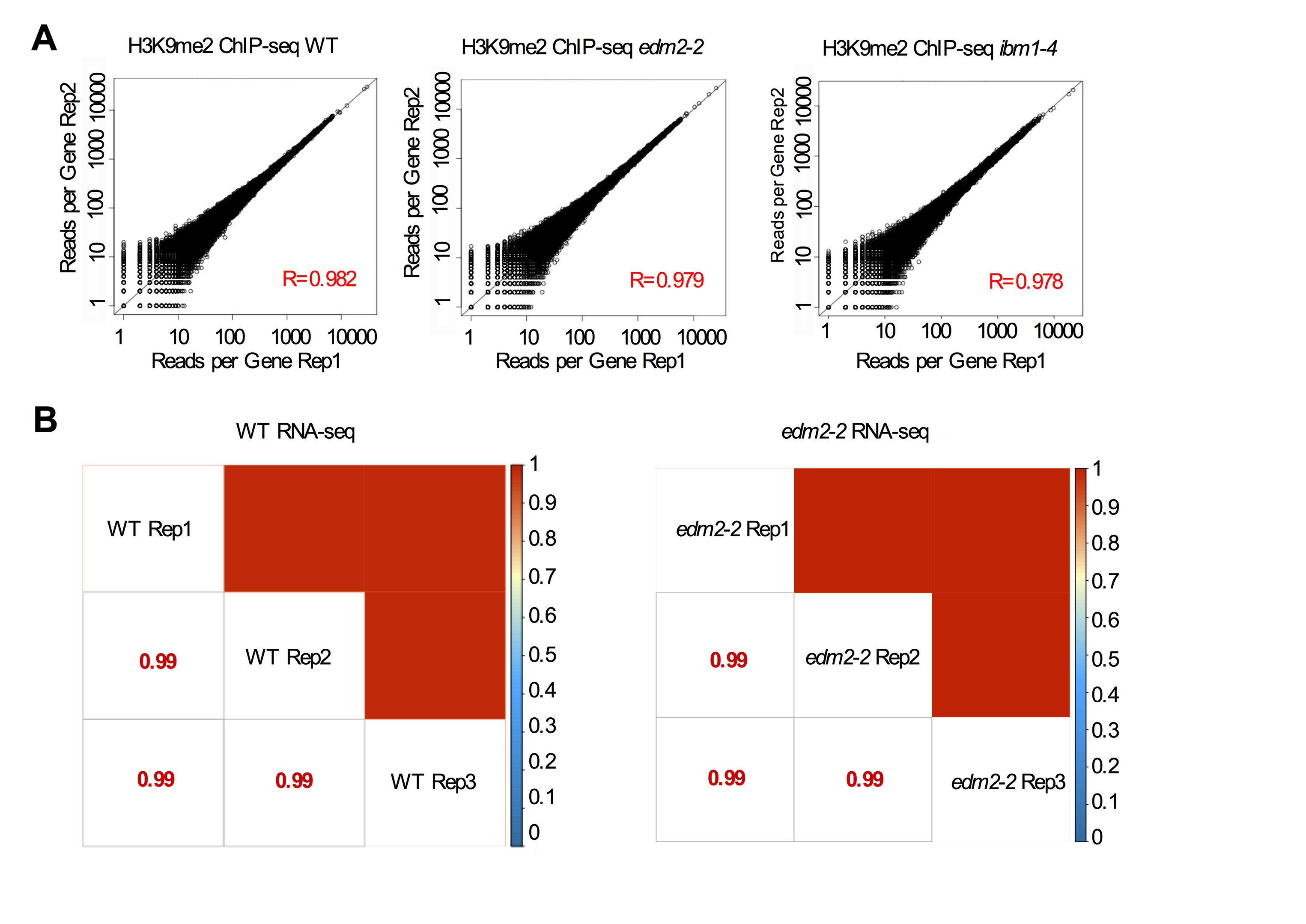

Supplement: S2 Fig — Spearman correlation for replicates of ChIP-seq (A) and RNA-seq (B) analyses. (TIF) [file pgen.1008993.s002.tif]

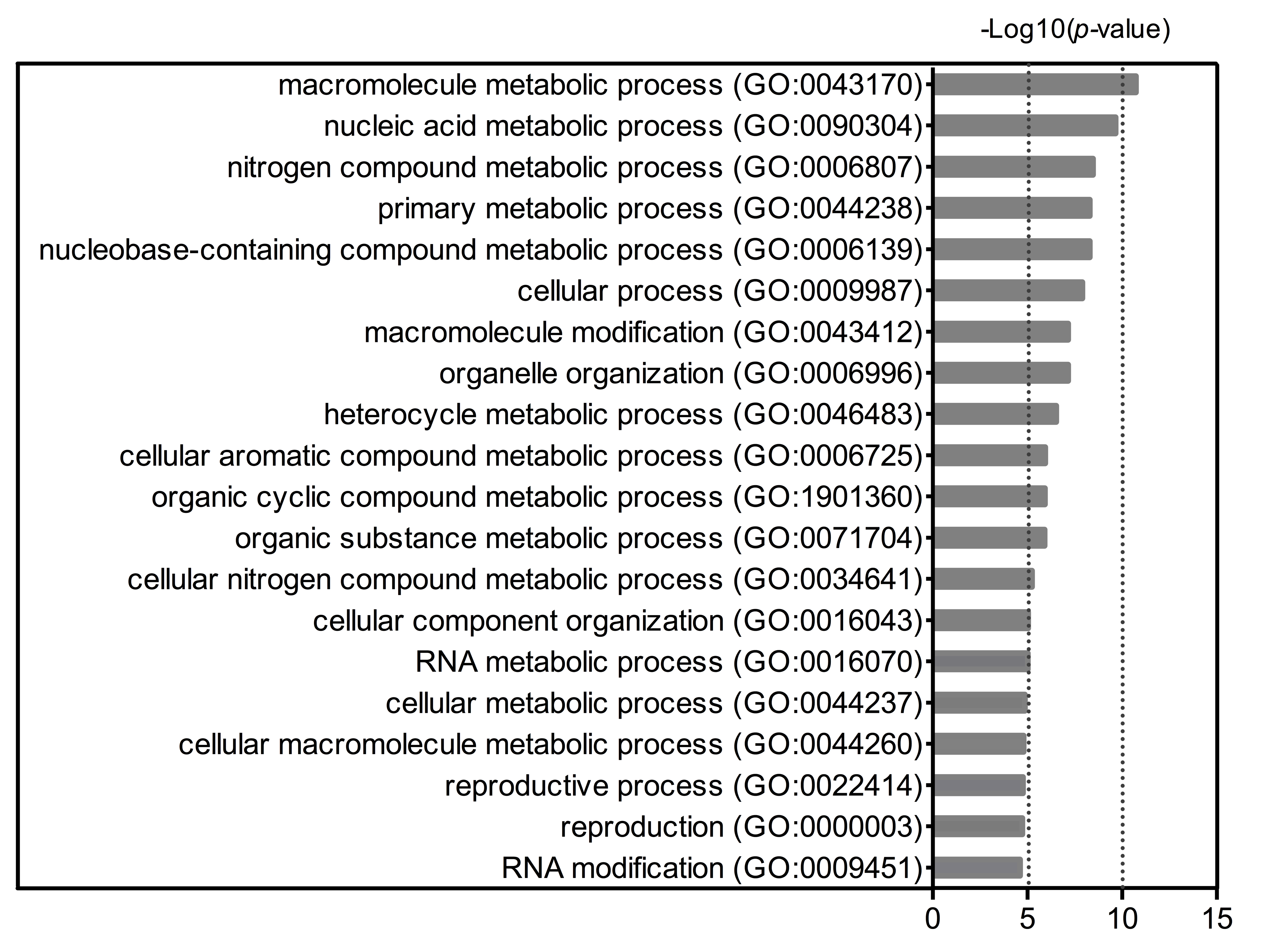

Supplement: S3 Fig — (TIF) [file pgen.1008993.s003.tif]

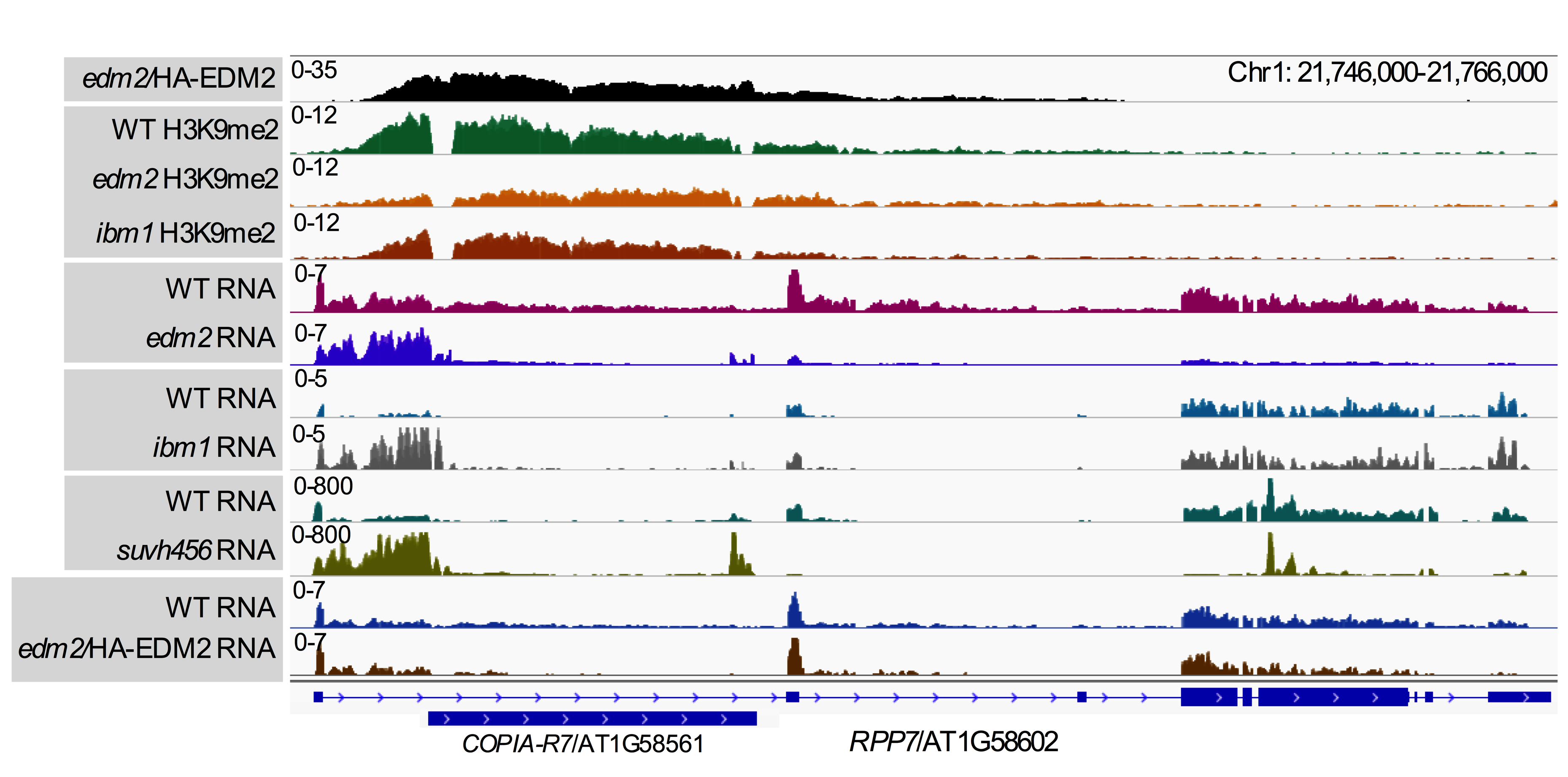

Supplement: S4 Fig — The y-axis represents coverage values (normalized per million mapped reads). (TIF) [file pgen.1008993.s004.tif]

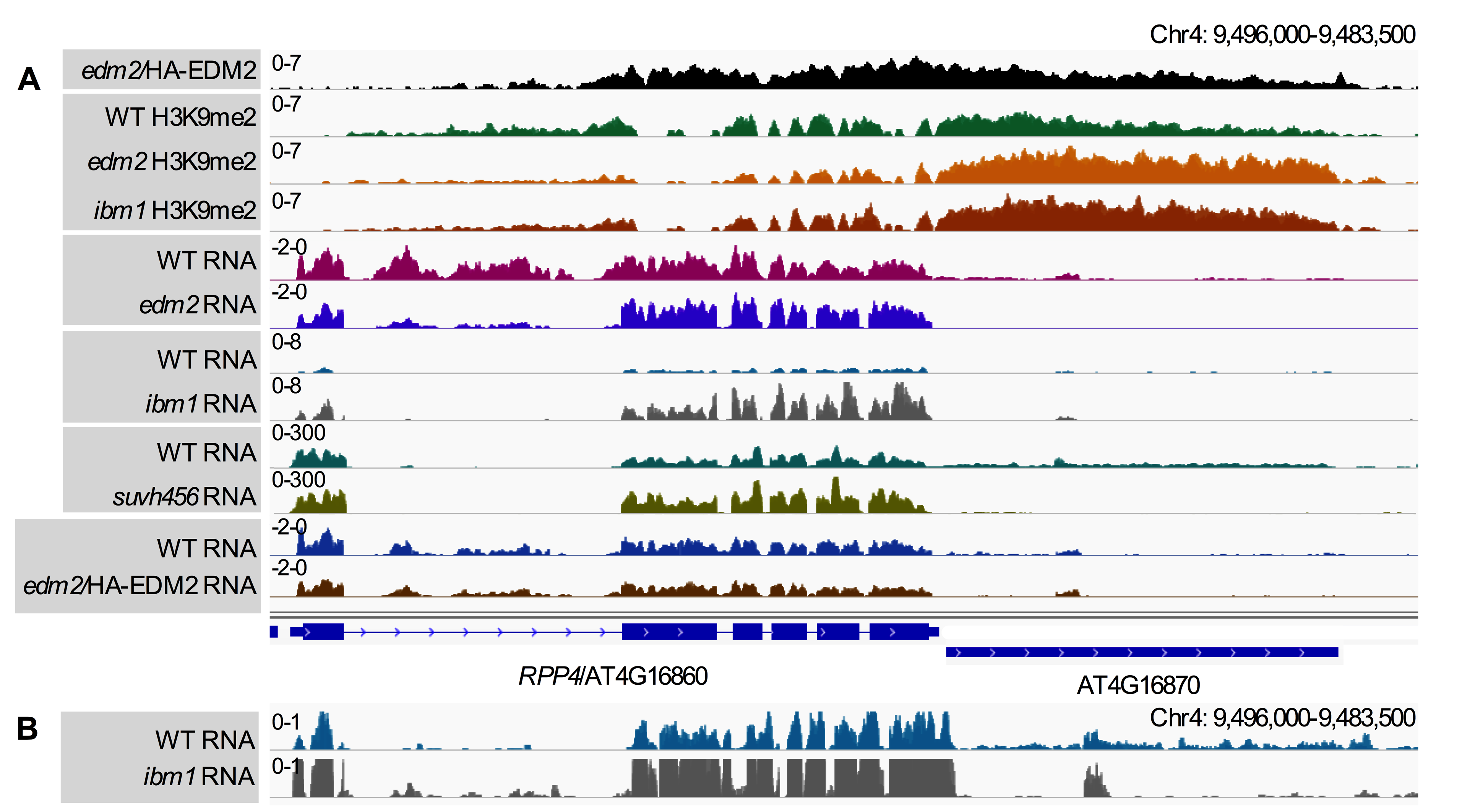

Supplement: S5 Fig — (A) Genome browser view of HA-tagged EDM2 ChIP-seq, H3K9me2 ChIP-seq and RNA-seq data at RPP4 locus. The y-axis represents coverage values (normalized per million mapped reads). (B) Genome browser view of WT and ibm1 RNA-seq data at RPP4 locus with different data ranges. (TIF) [file pgen.1008993.s005.tif]

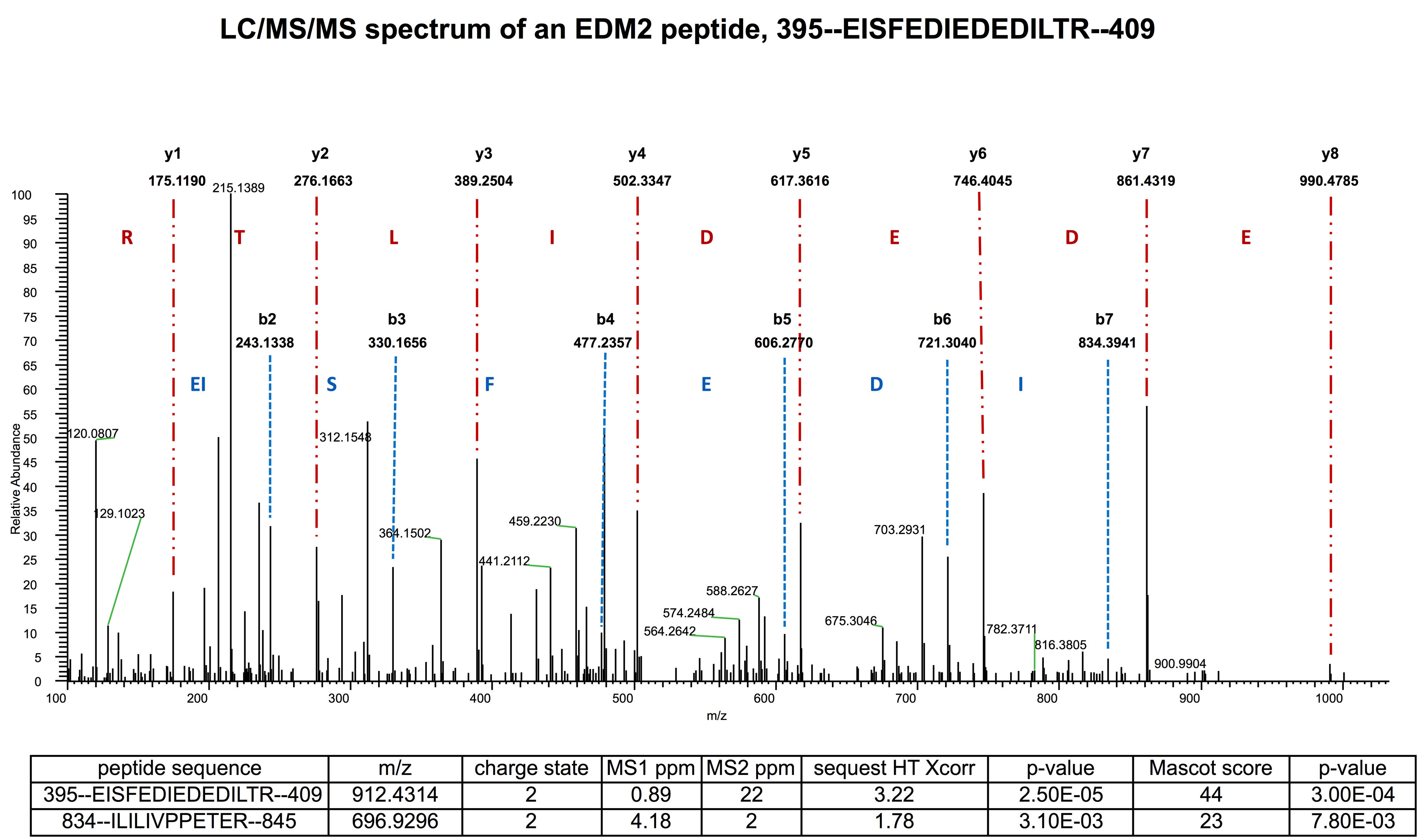

Supplement: S6 Fig — All detected y-series as well as b-series fragment ions are labeled. Many neutral-loss fragments are also detected but not labeled here. Both Sequest HT and Mascot search engines matched this spectrum to an EDM2 peptide, 395—EISFEDIEDEDILTR—409, with high confidence. Summary for two mass spectrometry identified EDM2 peptides are shown in the bottom. (TIF) [file pgen.1008993.s006.tif]

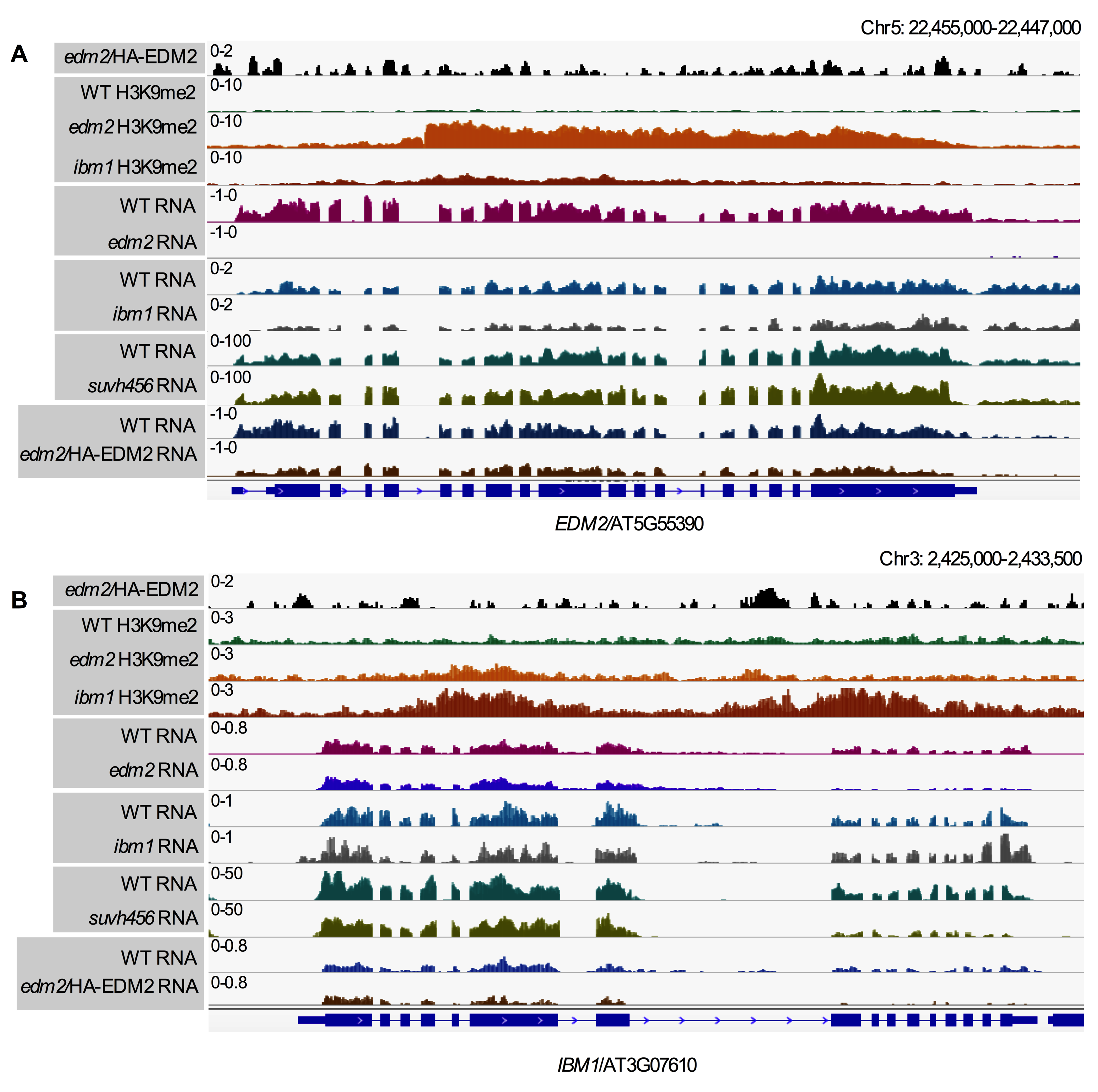

Supplement: S7 Fig — (A and B) Genome browser view of HA-tagged EDM2 ChIP-seq, H3K9me2 ChIP-seq and RNA-seq data at the EDM2 (A) and IBM1 (B) loci. The y-axis represents coverage values (normalized per million mapped reads). (TIF) [file pgen.1008993.s007.tif]

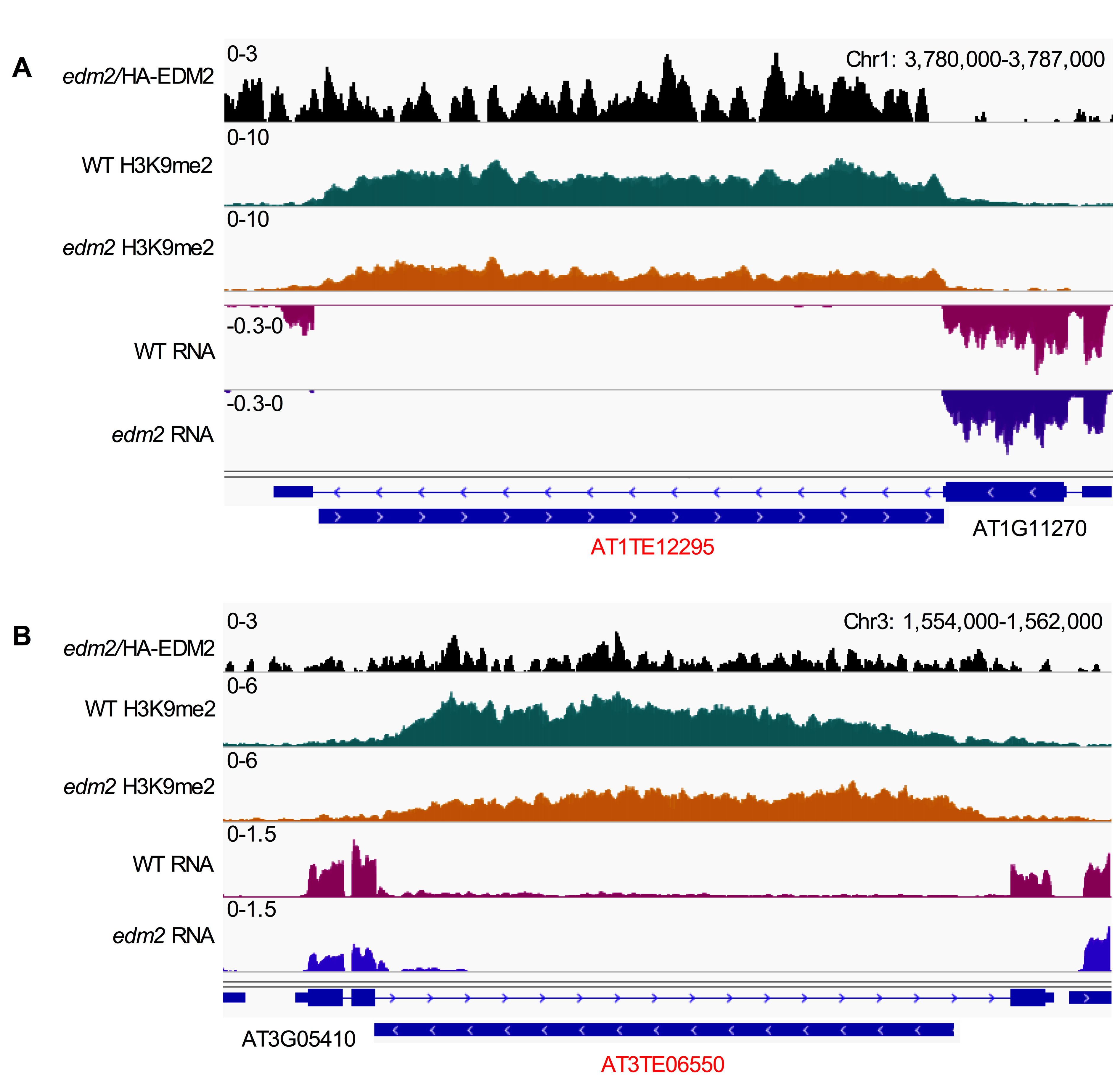

Supplement: S8 Fig — (A and B) Genome browser view of HA-tagged EDM2 ChIP-seq, H3K9me2 ChIP-seq and RNA-seq data at AT1G11270 (A) and AT3G05410 (B) loci. The y-axis represents coverage values (normalized per million mapped reads). (TIF) [file pgen.1008993.s008.tif]

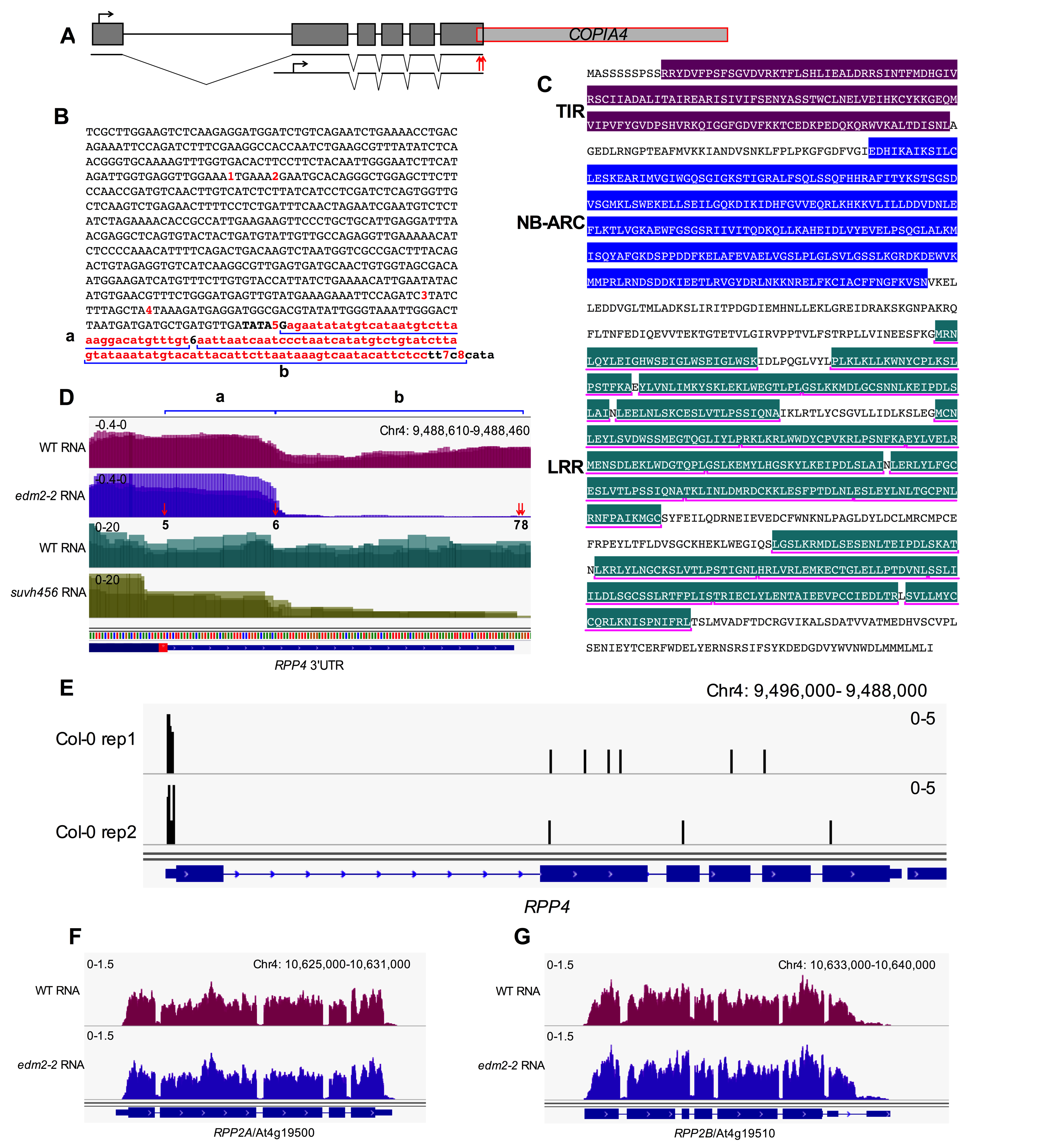

Supplement: S9 Fig — (A) Schematic representation of RPP4 with two alternative RNA transcript isoforms. (B) Nucleotide sequence of RPP4 exon 6. Coding nucleotides of exon 6 are in upper case black letters. Lower case letters in red are 3’UTR. The 130 bp of 5’LTR sequence for COPIA4 are shown in bold. Polyadenylation sites determined by 3’RACE are labeled by numbers 1–8. Blue underlined sequences between areas a (#5- #6) and b (#6- #8) indicate regions used for read counts shown in Fig 5B. (C) Amino acid sequence of protein isoforms encoded by RPP4. Purple: TIR domain; Blue: NB-ARC domain; Green: Leucine-rich repeat (LRR) domain, each one of 18 putative LRRs (predicted by Uniprot: F4JNA9) is underlined in pink. (D) Genome browser view of edm2, suvh456 and respective WT RNA-seq data at RPP4 3’UTR. Red arrows indicate polyadenylation sites 5–8 shown in (B). Blue underlined areas a (#5- #6) and b (#6- #8) indicate regions used for read counts shown in Fig 5B. Genome tracks of three biological replicates were overlaid and displayed in IGV. Each single replicate is represented by the lightest shade. Overlaps between two replicates are of medium darkness, while overlaps of all three replicates are of maximal darkness. (E) Genome browser view of TSSs at RPP4. Genome tracks of two biological replicates for Col-0 were shown. TSS-seq data were obtained from NCBI (GEO: GSE113677)[36]. (F and G) Genome browser views of edm2 and WT RNA-seq data at RPP2A (F) and RPP2B (G). (TIF) [file pgen.1008993.s009.tif]

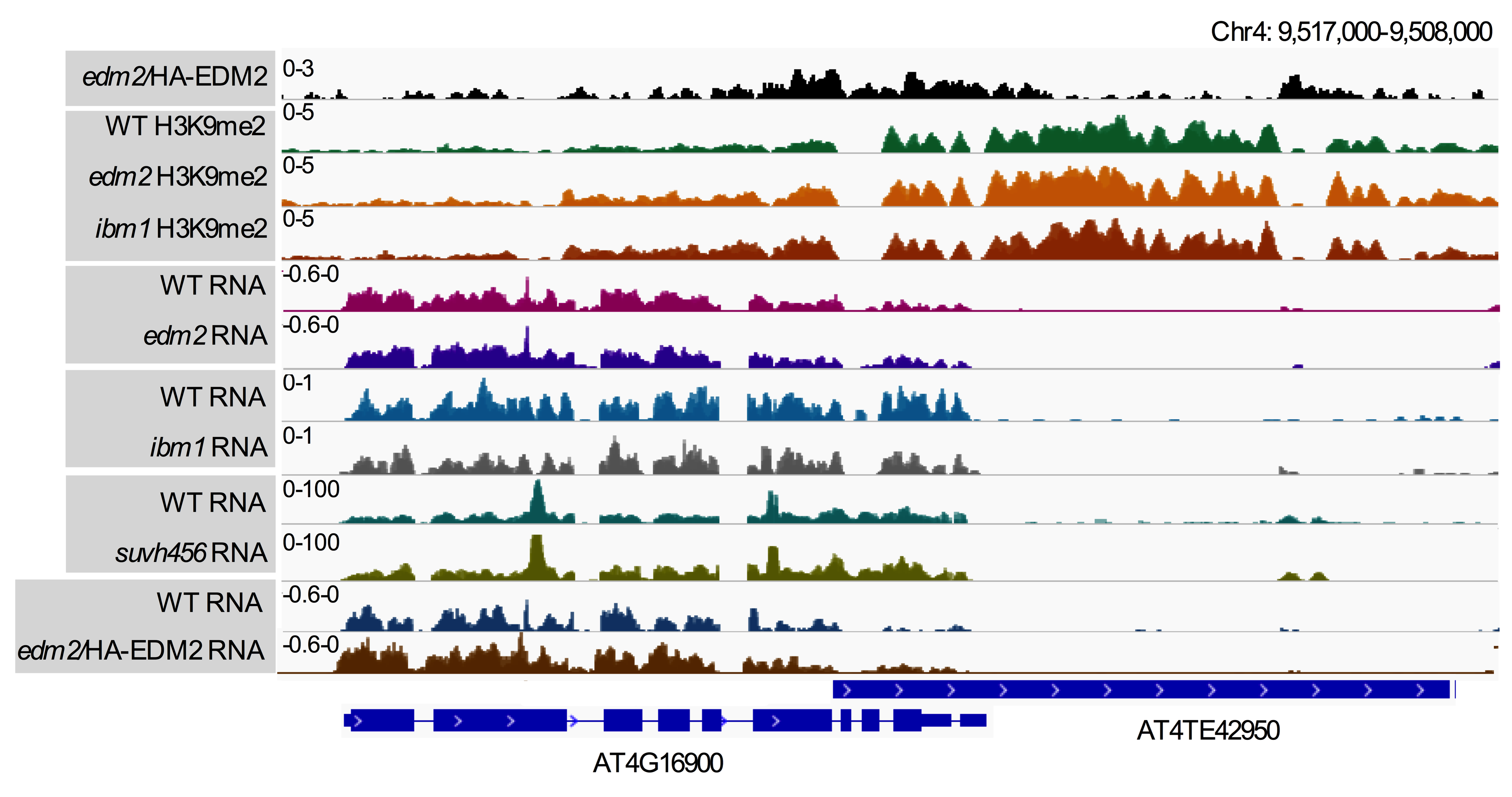

Supplement: S10 Fig — The y-axis represents coverage values (normalized per million mapped reads). (TIF) [file pgen.1008993.s010.tif]

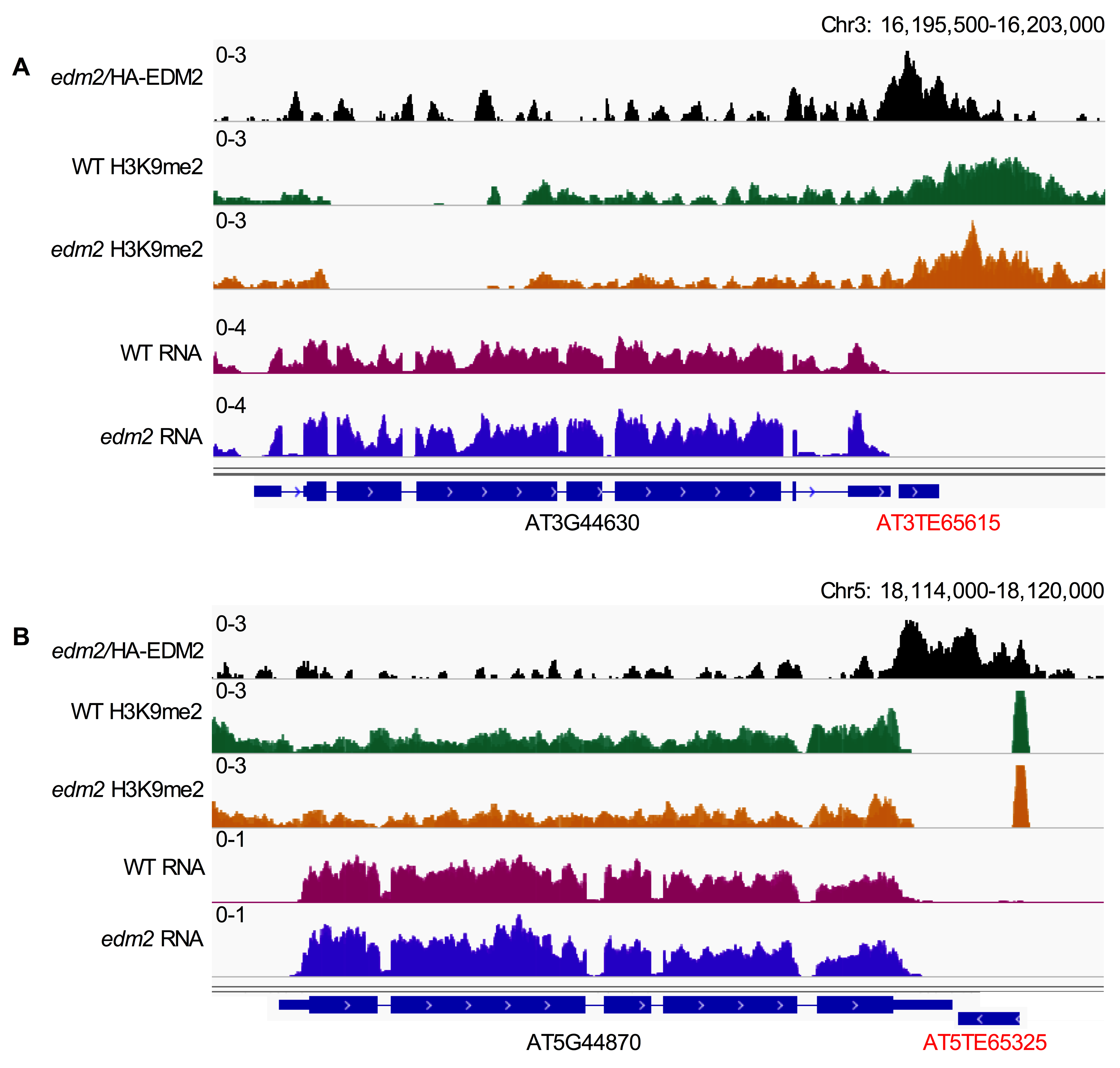

Supplement: S11 Fig — Genome browser view of HA-tagged EDM2 ChIP-seq, H3K9me2 ChIP-seq and RNA-seq at AT3G44630 and AT3TE65615 (A) and AT5G44870 and AT5TE65325 (B) loci. The y-axis represents coverage values (normalized per million mapped reads). (TIF) [file pgen.1008993.s011.tif]

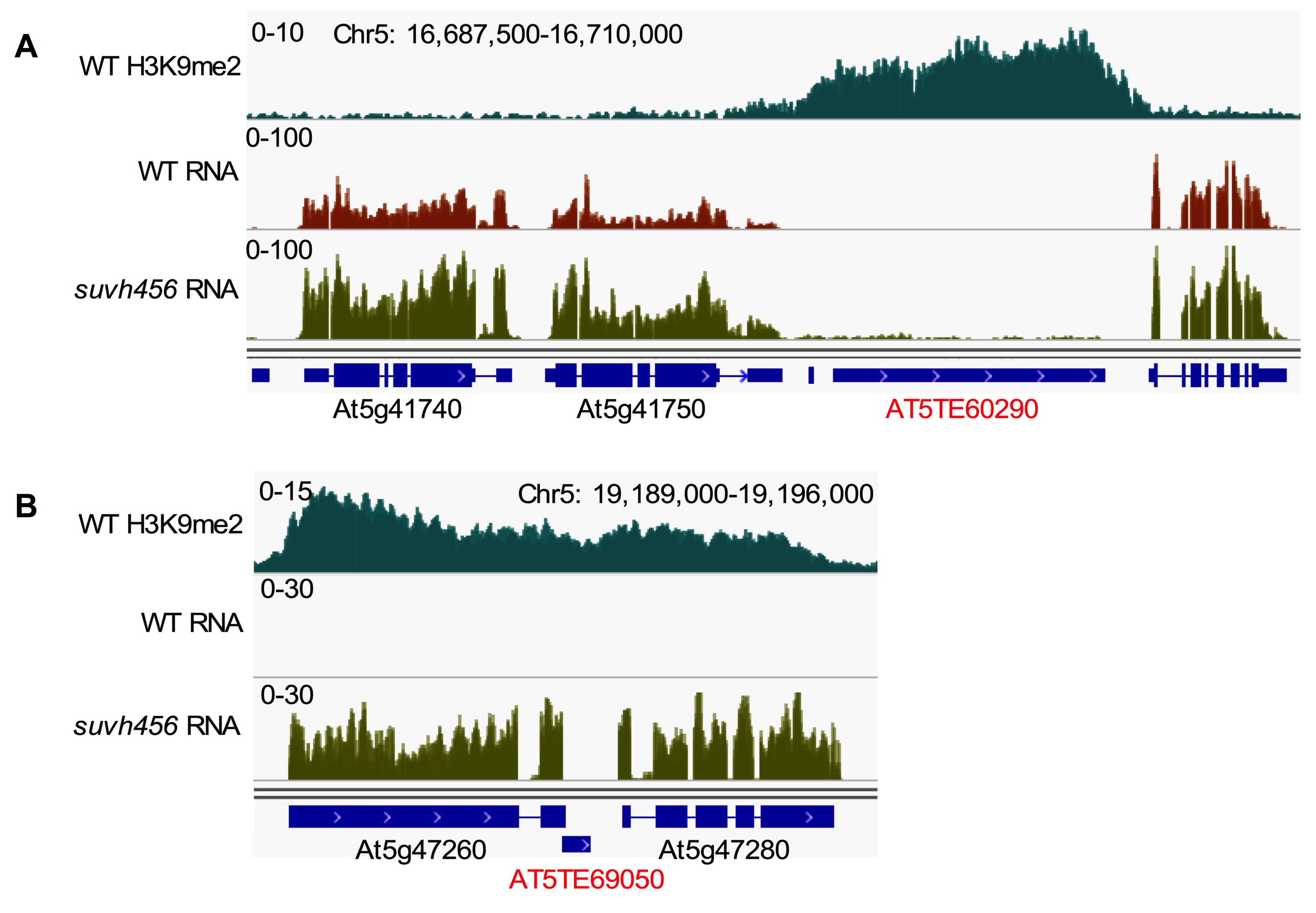

Supplement: S12 Fig — Genome browser view of AT5G41740 and AT5G41750 (A) and AT5G47260 and AT5G47280 (B). The y-axis represents coverage values (normalized per million mapped reads). (TIF) [file pgen.1008993.s012.tif]

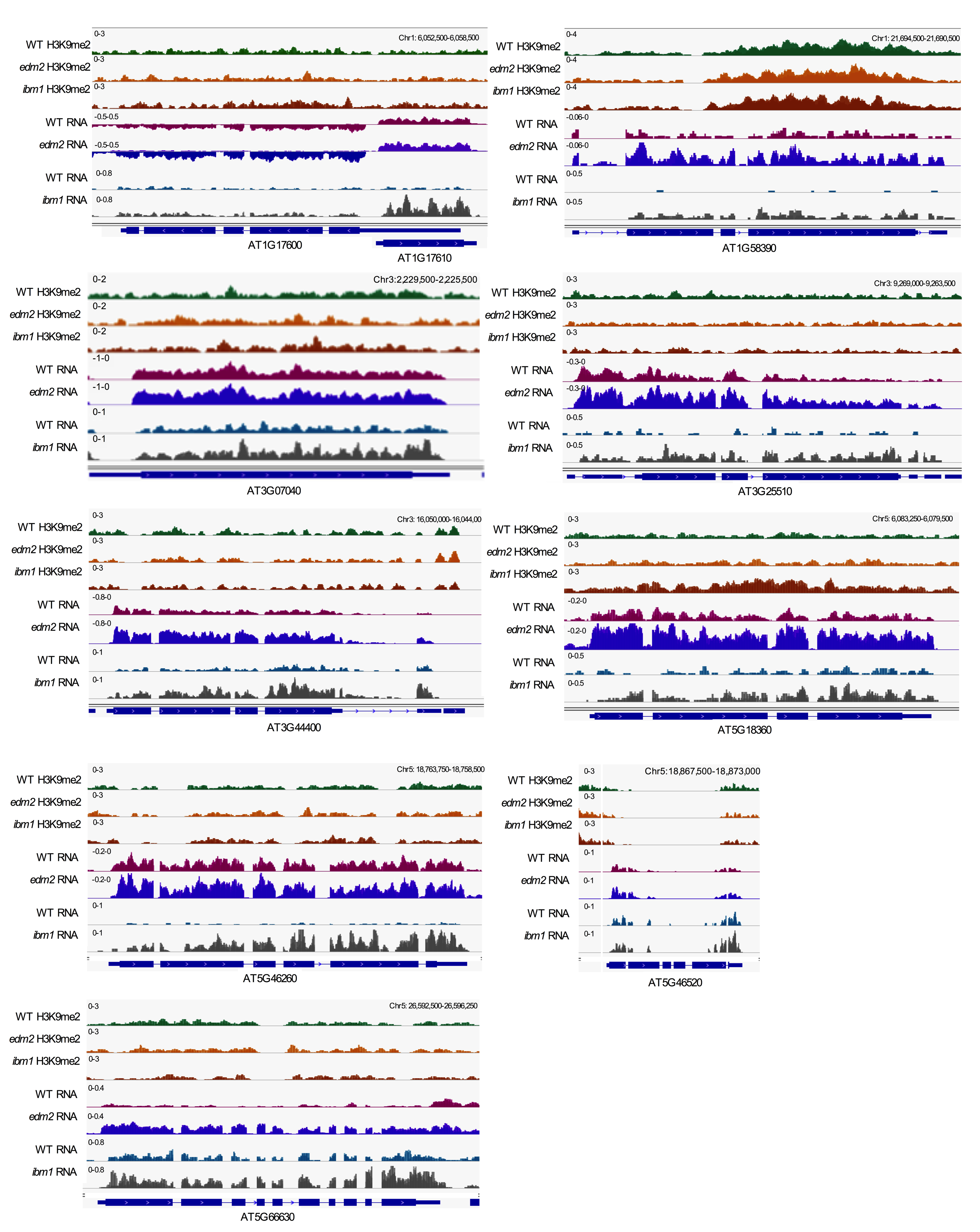

Supplement: S13 Fig — The AGI numbers for these loci are AT1G17600, AT1G17610, AT1G58390, AT3G07040, AT3G25510, AT3G44400, AT5G18360, AT5G46260, AT5G46520 and AT5G66630. The y-axis represents coverage values (normalized per million mapped reads). (TIF) [file pgen.1008993.s013.tif]

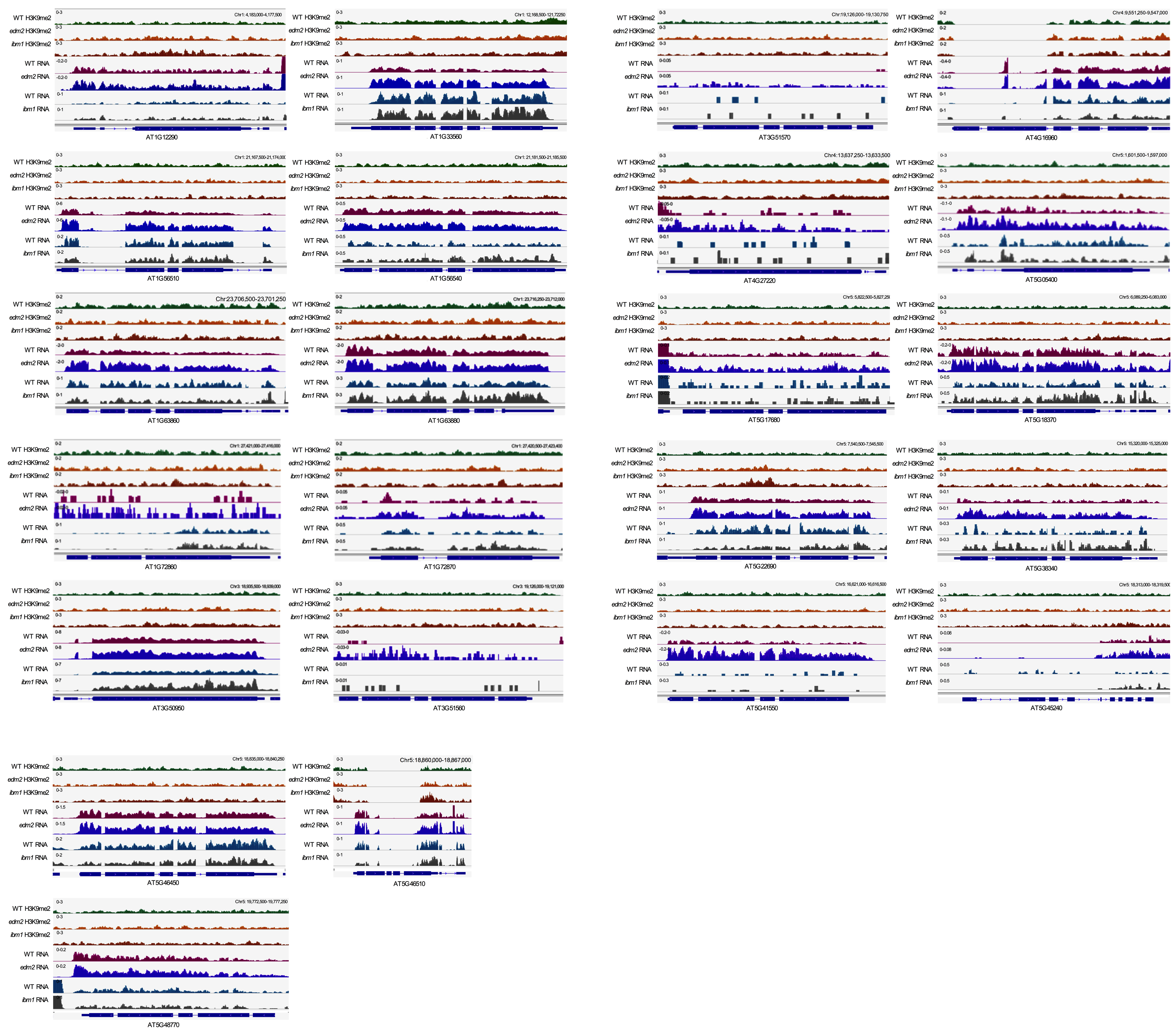

Supplement: S14 Fig — The AGI numbers for these loci are AT1G12290, AT1G33560, AT1G56510, AT1G56540, AT1G63860, AT1G63880, AT1G72860, AT1G72870, AT3G50950, AT3G51560, AT3G51570, AT4G16960, AT4G27220, AT5G05400, AT5G17680, AT5G18370, AT5G22690, AT5G38340, AT5G41550, AT5G45240, AT5G46450, AT5G46510 and AT5G48770. The y-axis represents coverage values (normalized per million mapped reads). (TIF) [file pgen.1008993.s014.tif]

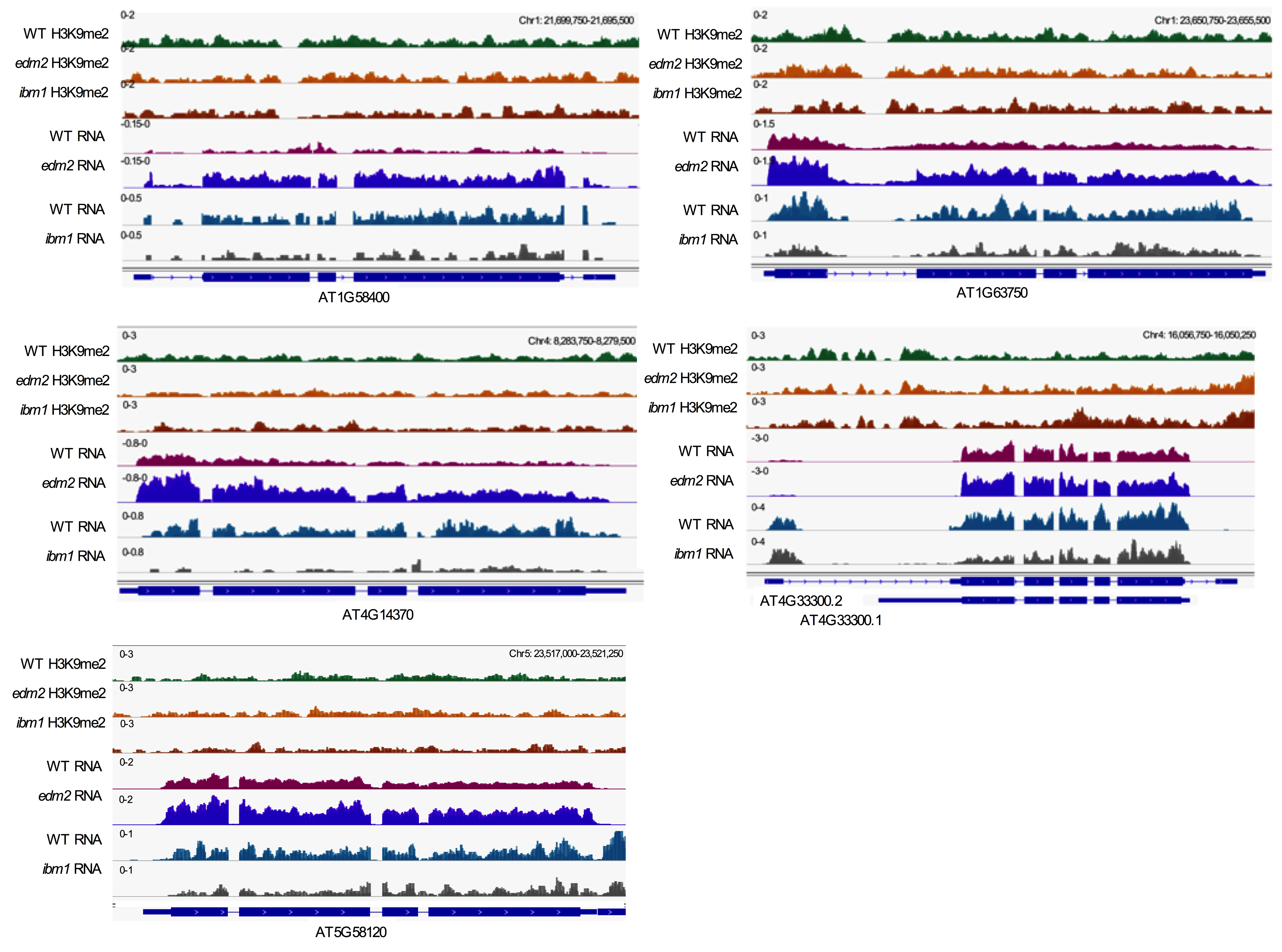

Supplement: S15 Fig — The AGI numbers for these loci are AT1G58400, AT1G63750, AT4G14370, AT4G33300 and AT5G58120. The y-axis represents coverage values (normalized per million mapped reads). (TIF) [file pgen.1008993.s015.tif]

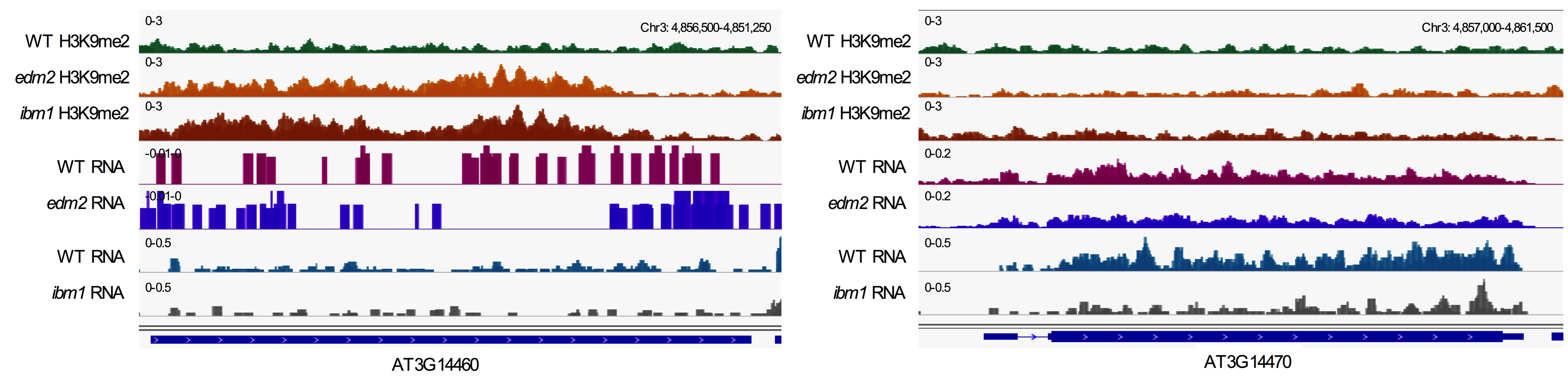

Supplement: S16 Fig — The AGI numbers for these loci are AT3G14460 and AT3G14470. The y-axis represents coverage values (normalized per million mapped reads). (TIF) [file pgen.1008993.s016.tif]

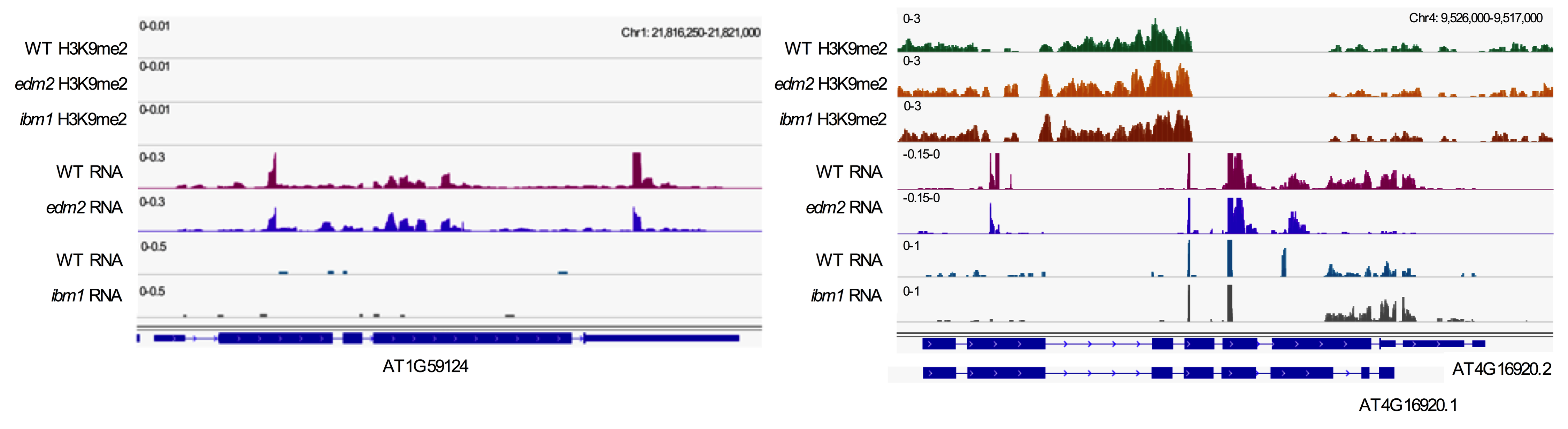

Supplement: S17 Fig — The AGI numbers for these loci are AT1G59124 and AT4G16920. The y-axis represents coverage values (normalized per million mapped reads). (TIF) [file pgen.1008993.s017.tif]

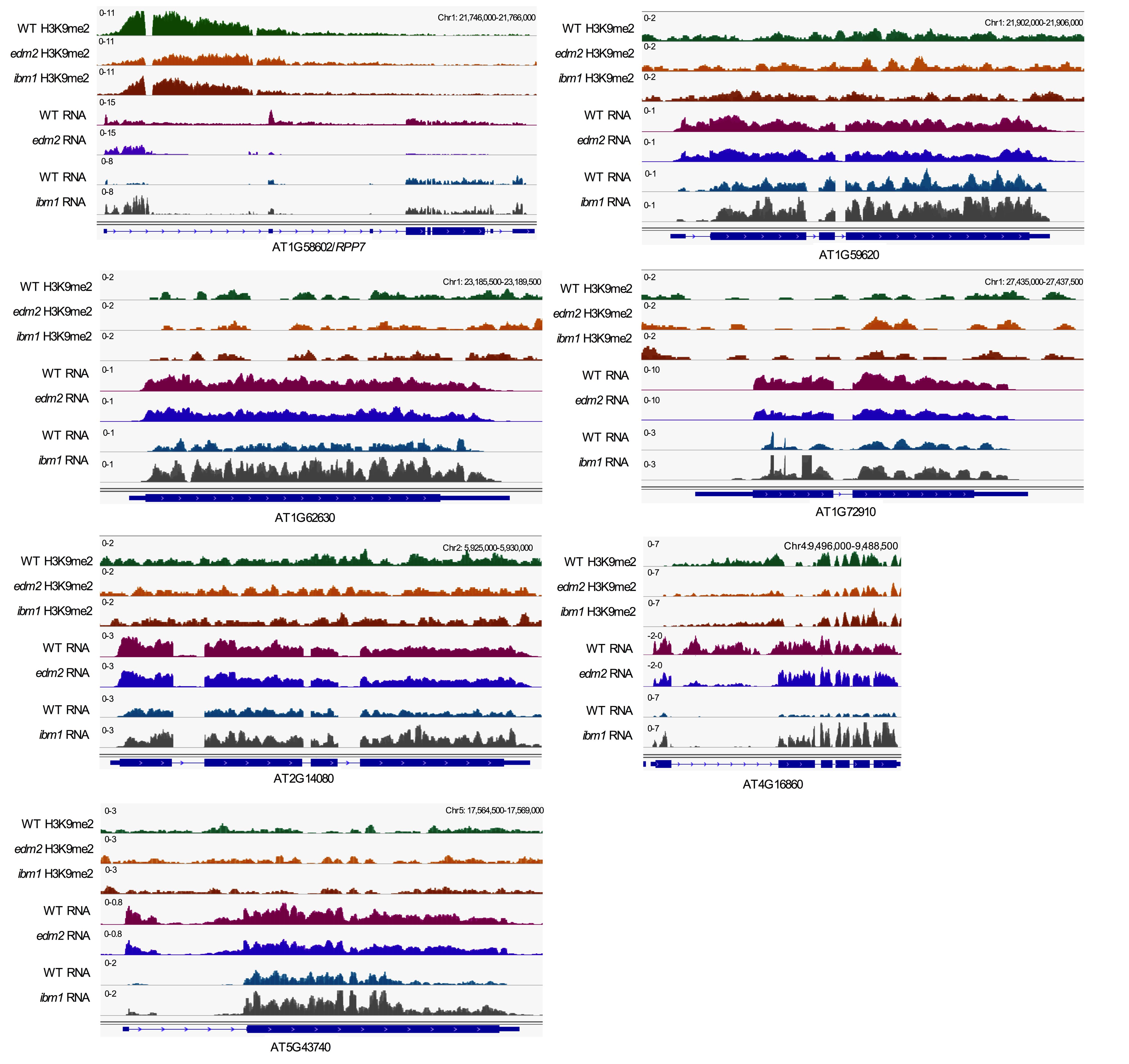

Supplement: S18 Fig — The AGI numbers for these loci are AT1G58602, AT1G59620, AT1G62630, AT1G72910, AT2G14080, AT4G16860 and AT5G43740. The y-axis represents coverage values (normalized per million mapped reads). (TIF) [file pgen.1008993.s018.tif]

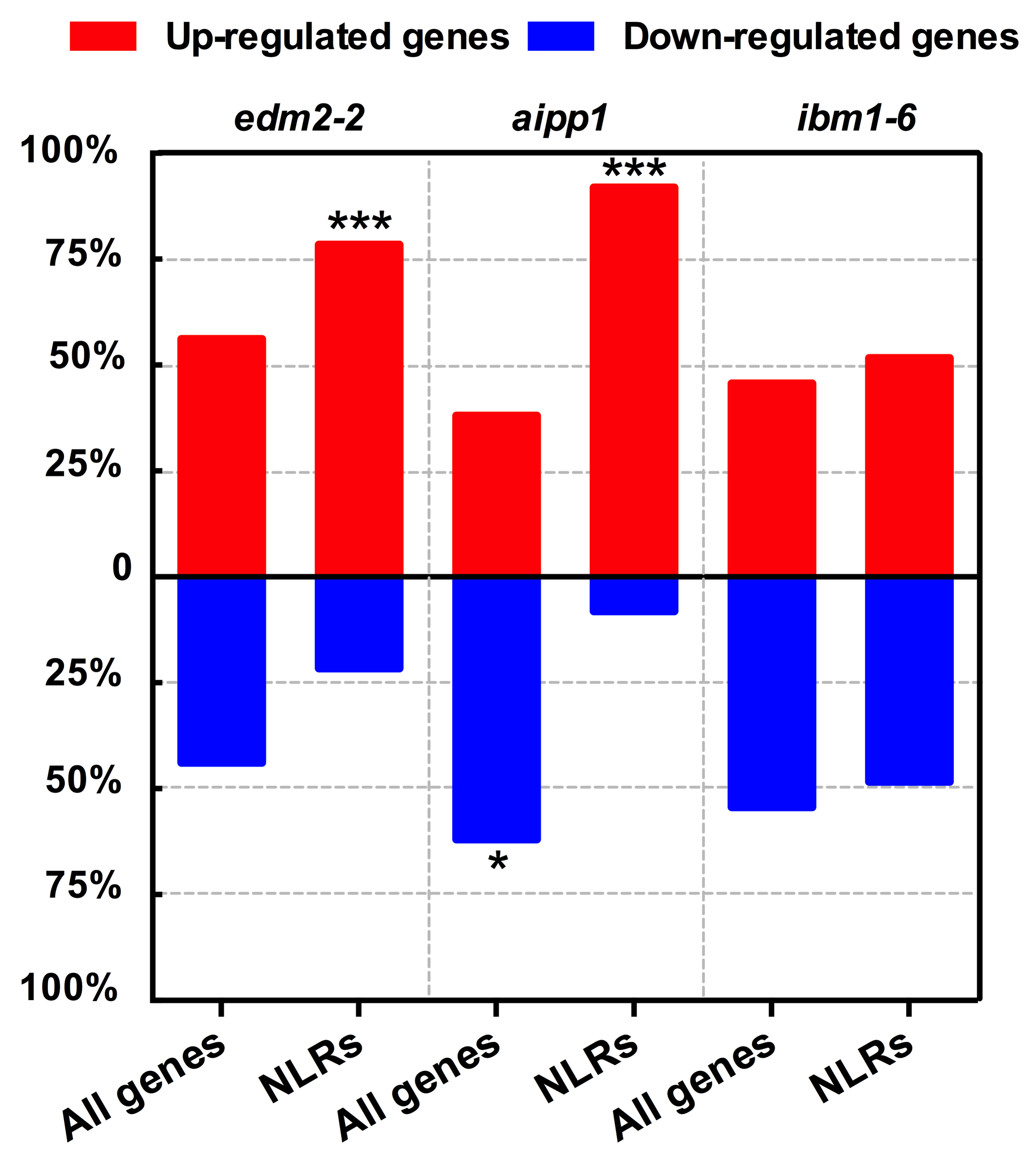

Supplement: S19 Fig — χ2 test of independence showed significant differences between actual and expected equal distribution (50% up-regulated and 50% down-regulated genes). *: P-value <0.05. ***: P-value <0.001. (TIF) [file pgen.1008993.s019.tif]

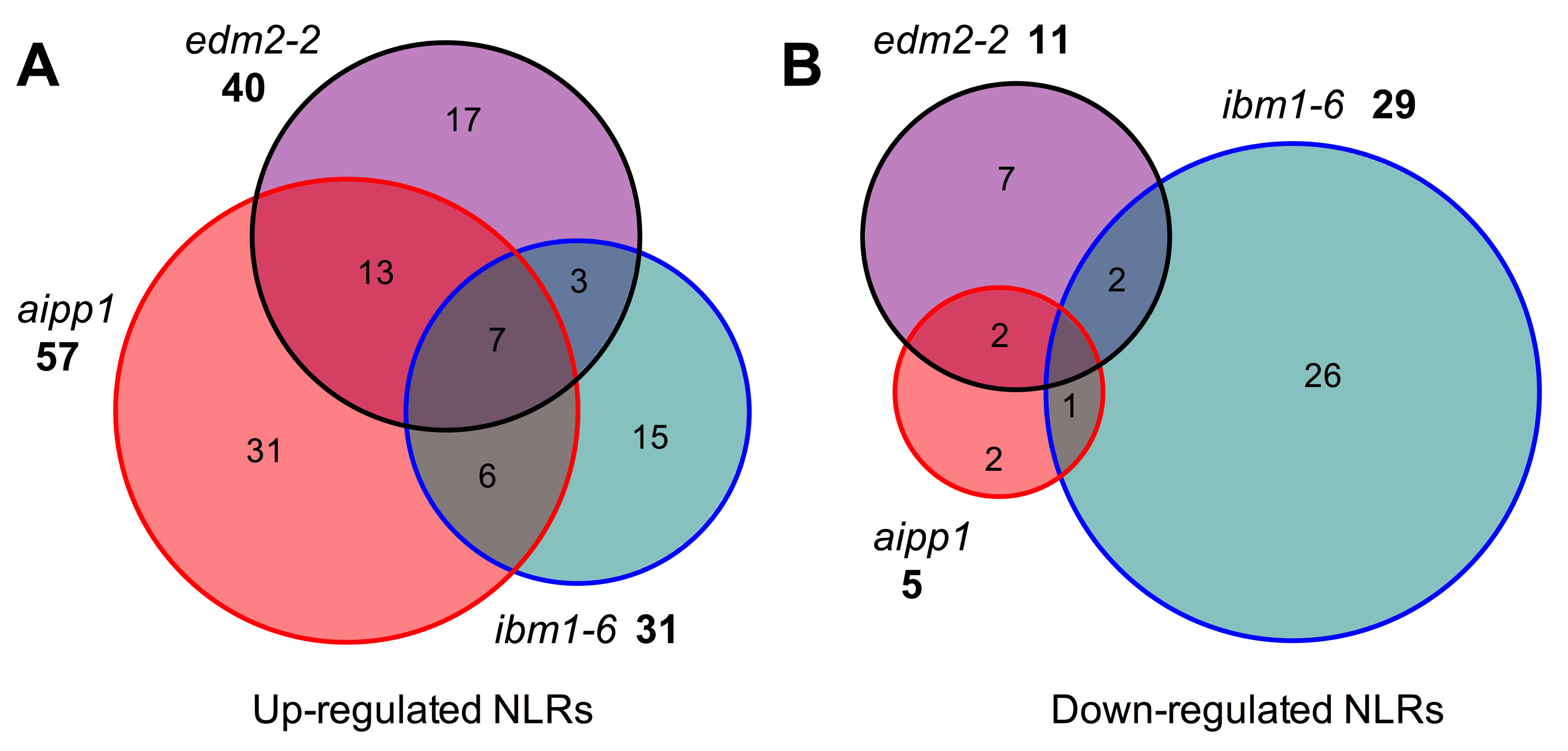

Supplement: S20 Fig — Venn diagram showing overlaps between edm2-2, aipp1 and ibm1-6 transcriptionally up-regulated (A) and down-regulated (B) NLR genes. (TIF) [file pgen.1008993.s020.tif]
